# Effects of combined tannic acid/fluoride on sulfur transformations and methanogenic pathways in swine manure

**DOI:** 10.1371/journal.pone.0257759

**Published:** 2021-09-23

**Authors:** Frederik Rask Dalby, Marcell Nikolausz, Michael Jørgen Hansen, Anders Feilberg

**Affiliations:** 1 Department of Engineering, Air Quality Engineering, Aarhus University, Aarhus N, Denmark; 2 Department of Environmental Microbiology, Helmholtz Centre for Environmental Research-UFZ, Leipzig, Germany; Aix-Marseille Universite, FRANCE

## Abstract

Livestock manure emits reduced sulfur compounds and methane, which affect nature and the climate. These gases are efficiently mitigated by addition of a tannic acid-sodium fluoride combination inhibitor (TA-NaF), and to some extent by acidification. In this paper, TA-NaF treatment was performed on swine manure to study the treatment influence on methanogenic pathways and sulfur transformation pathways in various laboratory experiments. Stable carbon isotope labeling revealed that both untreated and TA-NaF treated swine manures were dominated by hydrogenotrophic methanogenesis. However, in supplementary experiments in wastewater sludge, TA-NaF clearly inhibited acetoclastic methanogenesis, whereas acidification inhibited hydrogenotrophic methanogenesis. In swine manure, TA-NaF inhibited s-amino acid catabolism to a larger extent than sulfate reduction. Conversely, acidification reduced sulfate reduction activity more than s-amino acid degradation. TA-NaF treatment had no significant effect on methanogenic community structure, which was surprising considering clear effects on isotope ratios of methane and carbon dioxide. Halophile sulfate reducers adapted well to TA-NaF treatment, but the community change also depended on temperature. The combined experimental work resulted in a proposed inhibition scheme for sulfur transformations and methanogenic pathways as affected by TA-NaF and acidification in swine manure and in other inocula.

## Introduction

Livestock manure support a diverse microbial community, which drives the transformation of organic matter into volatile compounds that influence our environment and climate [1, 2]. Methane is a greenhouse gas formed primarily by demethylation of acetate or by reduction of carbon dioxide with hydrogen (H_2_) as electron donor [3, 4] in anaerobic environments such as in livestock manure. Reduced sulfur compounds are other end products of microbially degraded organic matter in livestock manure and these are particularly abundant in gas emissions from swine manure [5]. The most important ones are hydrogen sulfide, methanethiol, and dimethyl sulfide, which originate from sulfate reduction and amino acid catabolism [6–8]. These compounds contribute to odor [8, 9] and secondary aerosol sulfate [5]. Sulfur compound transformations and methanogenesis are closely related, as the microbes responsible for these processes compete for some of the same substrates. For example, acetate is used as carbon (C) source for acetoclastic methanogenesis and sulfate reduction [1, 10] with Gibbs free energy of reaction at standard conditions (ΔG°) of -31.0 kJ mol^-1^ [11] and -47.9 kJ mol^-1^ [12], respectively, indicating thermodynamic advantage for sulfate reducing bacteria [11]. Similarly, H_2_ is an electron donor in both hydrogenotrophic methanogenesis (ΔG° = -135.6 kJ mol^-1^) [13] and hydrogenotrophic sulfate reduction (ΔG° = -151.9 kJ mol^-1^) [10, 11]. Hydrogen is produced through e.g. syntrophic acetate oxidation, which is thermodynamically unfavorable at standard conditions with ΔG° = +104.1 kJ mol^-1^ [11, 14]. However, the reaction becomes feasible at low hydrogen partial pressure (<50 Pa), which can be reached by coupling the reaction to H_2_ consuming reactions such as hydrogenotrophic methanogenesis or hydrogenotrophic sulfate reduction [10, 15]. Despite the thermodynamic advantage of sulfate reducing bacteria, methanogens generally outcompete sulfate reducing bacteria in anaerobic digesters due to higher growth rates [10]. Additionally, methylotrophic methanogens are involved in sulfur and carbon cycling due to their ability to demethylate methanethiol to methane [16]. Methanethiol is a product of either hydrogen sulfide methylation or methionine degradation [8]. Considering these relationships, it is highly probable that manipulating or inhibiting one group of microbes affects the other and vice versa.

The detrimental effects of methane, reduced sulfur compounds and several other livestock gases on the climate and environment have raised the demand for innovating sustainable mitigation technologies. In recent studies, methane and reduced sulfur compounds emissions were significantly reduced from swine manure using tannic acid with sodium fluoride (TA-NaF) as inhibiting agent [17]. It was shown that inhibition with TA-NaF was of synergistic nature and it was proposed that the mode of inhibition was related to TA disrupting the cell membrane, which allows toxic fluoride to flow into the cell and inhibit metabolism [17]. Dalby et al. [18] studied isotope signatures of methane and carbon dioxide and suggested that acetoclastic methanogens were more susceptible to TA-NaF inhibition. However, isotope signatures are not conclusive evidence of inhibition of a specific pathway. The effect of TA-NaF on sulfur transformations has not been studied so far. Understanding the biochemical transformations and their interactions with commercial (e.g. sulfuric acid treatment) or newly developed mitigation agents is crucial when optimizing their applications. For example, a mitigating agent might be effective at reducing emission of methane by inhibiting the acetoclastic pathway, but less so if methane comes from reduction of carbon dioxide. A widely deployed technique to study such interactions is the usage of isotope-labeled precursors supported by microbial community analysis. The application of ^33^S-sulfate labeling in swine manure to examine sulfur transformations has recently been demonstrated and it was shown that approximately 80% of hydrogen sulfide came from sulfate reduction [7]. Likewise, the incorporation of ^13^C-labeling of the methyl carbon in acetate has been used in numerous studies to trace methanogenic pathway activity [19–21]. Labeling of the methyl carbon is advantageous because for acetoclastic methanogenesis the methyl carbon is known to end up in methane, whereas the carbon in carboxylic acid is converted to carbon dioxide [20]. Conversely, in the first step of syntrophic acetate oxidation coupled to hydrogenotrophic methanogenesis both carbon atoms in acetate are oxidized to carbon dioxide [20].

The aim of this study was to identify affected methanogenesis and sulfur transformation pathways upon TA-NaF amendment to swine manure. This was accomplished in isotope labeling experiments using proton-transfer-reaction mass spectrometry (PTR-MS) and cavity ring-down spectroscopy (CRDS). These analytical tools were supported by microbial community analysis targeting the 16S rRNA gene of bacteria and the mcrA gene of archaea. In some of the experiments, manure acidification with sulfuric acid or hydrochloric acid was also examined as a reference additive for reducing gas emissions. Similarly, some experiments were carried out on cattle manure and wastewater sludge to understand whether microbial community structure and substrate composition had an influence on the effect of TA-NaF. The following main objectives were addressed:

- Determine, which methanogenic pathway and methanogens dominate under the influence of TA-NaF inhibition in swine manure and other inocula.
- Determine the degree of inhibitory effect on sulfate reduction or cysteine and methionine degradation by TA-NaF and acidification treatment of swine manure.

Based on a recent study [17] and earlier observations by Ottosen et al. [22], the hypothesis was that TA-NaF would strongly inhibit methionine degradation, whereas acidification would inhibit sulfate reduction to a higher degree. We expected that both TA-NaF would primarily inhibit acetoclastic methanogenesis, leading to the dominance of hydrogenotrophic methanogenesis [18]. Dominance of hydrogenotrophic methanogenesis has repeatedly been observed in environments under chemical stress [23, 24].

## Materials and methods

**Fig 1:**
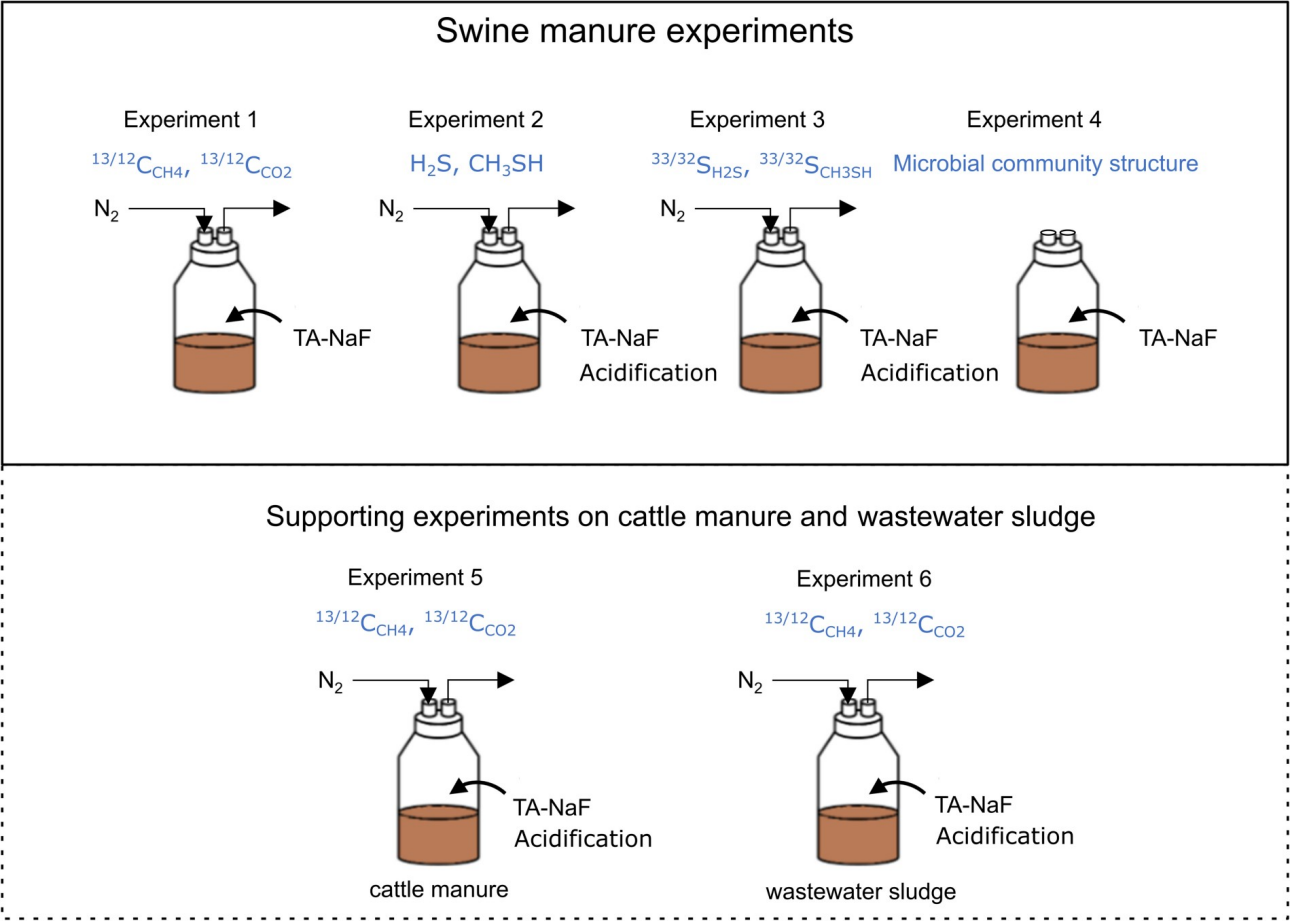
Overview of experiments conducted in this study. Blue text indicates the measured components. The inocula were treated with tannic acid with sodium fluoride (TA-NaF) or acidified with sulfuric acid or hydrochloric acid. Experiment 4 was carried out with both swine and cattle manure.

This study comprises experiments conducted on different swine manures treated with tannic acid combined with sodium fluoride (TA-NaF) or by acidification. While the treatment effects on different swine manures might limit direct comparison, it provides a fuller picture of the generic inhibition mode. For experiment 1, a lower TA-NaF dose was used to ensure that methanogenesis was not inhibited to the extent where methane would be unmeasurable, but at the same time would influence the methanogenesis activity. The experimental work is summarized in Fig 1 and details are shown in Table 1.

**Table 1:** Detailed experiment overview.

Measured components	Substrate	Treatment	Temp (°C)	n	δ^13^C_2-C-Ac_ *(*‰)	*R*_*ex*_(^33/32^S)_SO4_(%)
**Swine manure experiments**						
*Methanogenesis pathways–experiment 1, Fig 3*						
δ^13^C_CH4_, δ^13^C_CO2_	Swine manure 1	2.5:1 mM TA:NaF	23	4	7719 ± 451	0
CH_4_ production,	Swine manure 1	None	23	4	7719 ± 451	0
CO_2_ production	Swine manure 1	None	23	4	-28.5	0
*Reduced sulfur compounds production–experiment 2, Fig 4*						
H_2_S production	Swine manure 2	5:1 mM TA:NaF	23	3	-28.5	0
CH_3_SH production	Swine manure 2	H_2_SO_4_ acidification (pH 5.5)	23	3	-28.5	0
	Swine manure 2	None	23	3	-28.5	0
*Reduced sulfur compounds pathways–experiment 3, Fig 5*						
*R*(^33/32^S)_H2S_	Swine manure 3	5:1 mM TA:NaF	23	4	-28.5	5.3 ± 0.2
*R*(^33/32^S)_CH3SH_	Swine manure 3	HCl acidification (pH 5.5)	23	4	-28.5	5.3 ± 0.2
	Swine manure 3	None	23	4	-28.5	5.3 ± 0.2
	Swine manure 3	None	23	3	-28.5	0
*Microbial community structure–experiment 4, Figs 6 and 7*						
Microbial community structure	Swine manure 4	5:1 mM TA:NaF	23	2	-28.5	0
	Swine manure 4	None	23	2	-28.5	0
	Swine manure 4	5:1 mM TA:NaF	38	2	-28.5	0
	Swine manure 4	None	38	2	-28.5	0
	Cattle manure 1	5:1 mM TA:NaF	23	2	-28.5	0
	Cattle manure 1	None	23	2	-28.5	0
	Cattle manure 1	5:1 mM TA:NaF	38	2	-28.5	0
	Cattle manure 1	None	38	2	-28.5	0
**Supporting experiments with cattle manure and wastewater sludge**						
*S1 Appendix–experiment 5, S1 Fig*						
^13/12^R_CH4_, ^13/12^R_CO2_	Cattle manure 2	7.5:1 mM TA:NaF	23	3	27690 ± 818	0
CH_4_ production,	Cattle manure 2	H_2_SO_4_ acidification (pH 5.5)	23	3	27690 ± 818	0
CO_2_ production	Cattle manure 2	None	23	4	27690 ± 818	0
*S2 Appendix–experiment 6, S2 Fig*						
^13/12^R_CH4_, ^13/12^R_CO2_	Wastewater sludge	5:1 mM TA:NaF	23	2	5152 ± 355	0
CH_4_ production,	Wastewater sludge	HCl acidification	23	2	5152 ± 355	0
CO_2_ production	Wastewater sludge	None	23	2	5152 ± 355	0
	Wastewater sludge	None	23	2	-28.5	0

δ^13^C_2-C-Ac_ refers to the isotope ratio on the methyl carbon in acetate using delta notation. Where ^13^C-acetate was not added to manures δ^13^C_2-C-Ac_ is assumed to be -28.5 ‰. Rex(33/32S)SO4 is the excess isotope ratio between ^33^S and ^32^S in sulfate.

### Experimental setup for gas measurements

**Fig 2:**
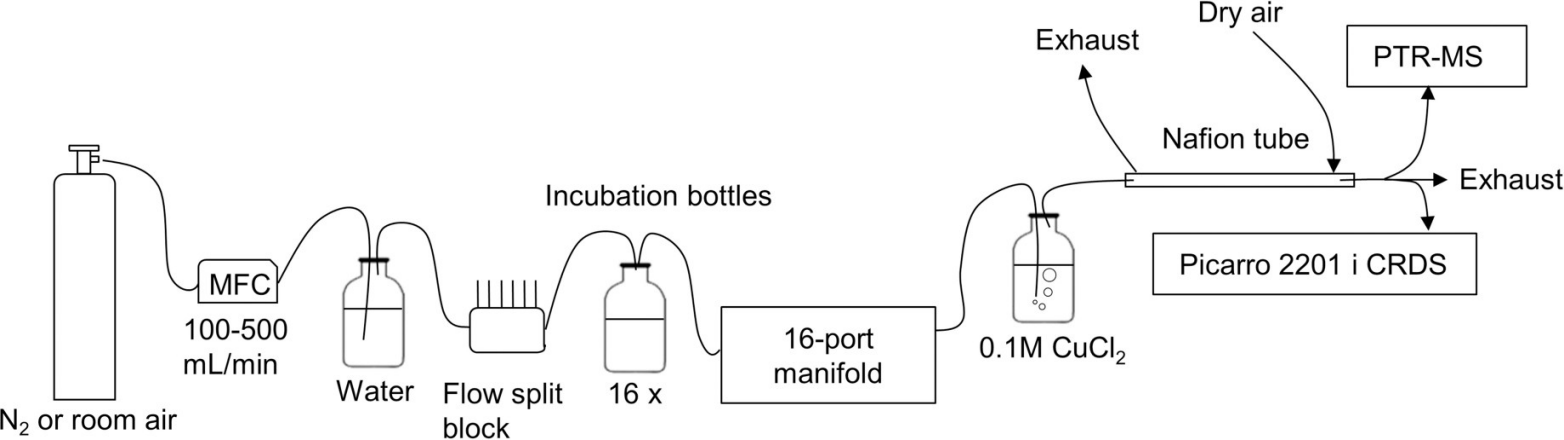
Gas measurement setup with incubation experiments. MFC is mass flow controller; PTR-MS is proton transfer reaction mass spectrometry; CRDS is cavity ring-down spectroscopy.

A headspace gas monitoring setup shown in Fig 2 was used in all experiments except experiments that examined microbial community structure (experiment 4). A mass flow controller (MFC) (Bronkhorst EL-FLOW, Ruurlo, Netherlands) continuously dosed 100–500 mL min^-1^ of nitrogen or pressurized room air through a PTFE block distributing the flow to the headspaces of up to 16 incubation bottles containing inocula. An impinger flask filled with distilled water was inserted before the PTFE distribution block to humidify the dry nitrogen and prevent desiccation of the inocula. The incubation bottles outlets in the lids were directed to a 16-port distribution manifold (Picarro, Santa Clara, CA, USA), that switched between the incubation bottles every 15^th^ min. Consequently, nitrogen was only flowing through the incubation bottle headspace when the manifold port was open for that particular incubation bottle. For experiments that did not involve measurement of reduced sulfur compounds the manifold port outlet directed the gases emitted from the incubation bottles to a 0.1 M aqueous CuCl_2_ scrubber solution and further onwards through a Nafion tube (length 90 cm, inner diameter 1.1 mm) with a continuous counter air flow of approximately 3 L min^-1^ to remove cross-interfering compounds as described previously [21]. Methane and carbon dioxide isotopologues (^12^CH_4_, ^13^CH_4_, ^12^CO_2_, ^13^CO_2_) were measured with a cavity ring-down spectrometer (Picarro, Santa Clara, CA, USA) throughout the experimental period. For experiments involving measurement of reduced sulfur compounds the aqueous CuCl_2_ scrubber solution was removed from the setup and reduced sulfur compounds were measured with a proton-transfer-reaction mass spectrometer (PTR-MS) (Ionicon Analytik, Innsbruck, Austria).

### Incubation and isotope labeling of swine manure

#### Methanogenesis pathways—Experiment 1

Swine manure was collected at Aarhus University Pig Production Facility (Foulum, Denmark) from a pre-storage tank. The manure was sampled into 10 L PTFE containers and stored at room temperature for more than 5 months. Before experiment start the manure was sieved through 1.2 mm pores to remove larger particles and straw. Samples for manure characterization were collected and stored at -18°C until analysis. The pH of the bulk manure was measured with a pH-meter (Portamess 911, Knick, Germany). While agitating the bulk manure with a magnet stirrer (MR 3001 K, Heidolph, Germany), 100 g of sieved swine manure was added to each of 12 x 100 mL DURAN bottles. This ensured even particle distribution in the manure while pouring it into the incubation bottes. Subsequently, four of the manure bottes (DURAN incubation bottes with manure) were treated with tannic acid (Merck, CAS 1401-55-4) and sodium fluoride (Merck, CAS 7681-49-4) to final concentrations of 2.5 mM and 1 mM, respectively and the remaining eight manure bottles were not treated. Then 2.15 mL of 1.67M Sodium acetate (Merck, CAS 127-09-3, anhydrous > 99%) stock solution and 0.25 mL of 1.67M Sodium acetate-2-^13^C (Merck, CAS 13291-89-9, 99 atom % ^13^C) stock solution were added as substrates to all four treated bottles and to four untreated bottles. The remaining four untreated bottles were only amended with 2.40 mL of the unlabeled 1.67M Sodium acetate stock solution. The swine manure reactors were then incubated at 23°C in the experimental setup shown in Fig 2. The nitrogen flow through the reactor headspaces were initially set to 100 mL min^-1^ and then increased to 350 mL min^-1^ after two days till experiment end after 14 days. The flow adjustment was done to stay within the methane and carbon dioxide operational range of the CRDS analyzer. The ^13/12^C isotope ratios of methane and carbon dioxide as well as the production of methane and carbon dioxide were measured throughout the experiment.

#### Reduced sulfur compounds production—Experiment 2

Swine feces and urine were collected separately from three fattening pigs placed in metabolism cages at Aarhus University Pig Production Facility (Foulum, Denmark). The swine urine was tapped from the bladders through a catheter into 10 L PTFE containers and the produced feces was collected from a tray underneath the metabolism cages. The feces and urine was no more than 6 hours old before it was put at -18°C until experiment start. Before experiment start the urine and feces were thawed over 20 minutes in a water bath at 38°C. Then 10 g thawed swine feces and 30 g thawed swine urine was added to each of 9 x 100 mL DURAN incubation bottles, which were then thoroughly mixed with a spatula. Three of the bottles were then treated with tannic acid (Merck, CAS 1401-55-4) and sodium fluoride (Merck, CAS 7681-49-4) to final concentrations of 5 mM and 1 mM, respectively and three bottles were treated with 1 M sulfuric acid stock solution to pH 5.5. Three bottles were not treated. The bottles were incubated into the experimental setup shown in Fig 2. On a daily basis, one gram swine feces and three gram swine urine were added as additional substrate to the manure reactors. In addition, TA-NaF or sulfuric acid was added to the manure reactors on a daily basis to maintain the inhibitor concentrations of 5:1 mM TA:NaF or the pH at 5.5. The headspace exchange gas was pressurized room air dosed with a flow rate of 0.5 L min^-1^ per reactor independent of the valve position, meaning that gases were not accumulating in the headspace of each reactor. The experiment was conducted without the CuCl_2_ scrubber and Nafion tube for two reasons; 1) The CuCl_2_ scrubber removes reduced sulfur compounds and, 2) the CRDS was not used in this experiment, therefore it was not necessary to remove compounds that are known to cause spectral interference.

#### Reduced sulfur compounds pathways—Experiment 3

Swine manure was collected from a storage tank at Aarhus University Swine Production Facility (Foulum, Denmark). The manure was stored at 5°C in closed 10 L PTFE containers for 8 months and then sieved through 1.2 mm pores. Samples for sulfate concentration analysis were taken before experiment start and measurement of the bulk manure was done with a pH-meter (Portamess 911). Forty grams of the sieved swine manure was added to each of 15 x 100 mL DURAN reactors. The manure bottles were then treated with tannic acid with sodium fluoride to final concentrations of 5 mM and 1 mM, respectively, or treated with 0.5 M hydrochloric acid to a pH of 5.5. In addition, the manure bottles were amended with 17 mg sodium sulfate, (Merck, CAS 7757-82-6) and 0.6 mL sodium sulfate-^33^S (Merck, PubChem ID 329763644, 98%) from a 20 mM stock solution. Finally, all manure bottles were put into the experimental setup in Fig 2. The CuCl_2_ scrubber was removed from the setup to allow reduced sulfur compounds to be detected by a high sensitivity proton-transfer-reaction mass spectrometer (PTR-MS) with a quadrupole mass separator (Ionicon Analytik, Innsbruck, Austria). A PTR-MS with a time of flight mass analyzer, PTR-TOF4000 (Ionicon Analytik, Innsbruck, Austria), was used to examine potential cross-interference on nominal mass to charge (*m/z)* ratios. The cavity ring-down spectrometer shown in Fig 2 was not used in this experiment.

#### Microbial community structure—Experiment 4

Swine manure was collected from a local farmer near Leipzig, Germany and stored at 5°C for approximately one month until experiment start. In addition and for comparative purposes, cattle manure was collected from a storage tank of a biogas plant at the Deutsches Biomasseforschungszentrum, Leipzig, Germany. Before starting the experiment, the manure was diluted to equal volatile solids (VS) contents of 2% (w/w). Diluted swine manure or cattle manure in amounts of 120 g were added to 16 x 200 mL serum flasks. Four swine and four cattle manure flasks were then treated with tannic acid with sodium fluoride to final concentrations of 5 mM and 1 mM and the remaining eight flasks (four swine and four cattle) were left untreated. The serum flask headspaces were flushed with nitrogen prior to incubating all treatments in duplicates at both 23°C and 38°C (using a heating chamber). Since microbial community changes occur gradually and relatively slowly compared to gas production, 38°C was chosen as an additional incubation temperature to speed up microbial community adaptation. All 16 flasks were flushed with nitrogen every 2–3 day to minimize potential product inhibition and effects of biogas pressure buildup in the headspace on methanogenesis during the experimental period, which lasted 30 days. Samples for microbial community analysis and characterization of manure components were taken at experiment start and after 30 days and stored at -18°C until analysis.

#### Supporting experiments

Additional experiments on cattle slurry and wastewater sludge were carried out to ensure that the methods used were applicable to other substrates than swine manure, and to better understand how substrate type and origin affect microbial pathways. In the supporting experiments only methanogenesis was investigated. In general the supporting experiments were carried out in with similar methods as to experiment 1 where methanogenesis pathways were examined in swine manure. A detailed description is provided in S1 Appendix for cattle slurry (experiment 5), and in S2 Appendix for wastewater sludge (experiment 6).

### Gas analysis

#### Methane and carbon dioxide

The cavity ring-down spectrometer was a Picarro G2201-i analyzer (Picarro, Santa Clara, CA, USA) running at a cavity pressure and temperature of 148 Torr and 45°C, respectively. The instrument measured in the iCH_4_-iCO_2_ dynamic measuring mode at a frequency of 0.2 Hz. The raw HR iCH_4_, raw HR CH_4_, raw iCO_2_, and raw CO_2_ outputs were used in the data processing. In experiment 1, 5, and 6, each time the accumulated gas in the headspace of each reactor was released by the valve, the production of methane and carbon dioxide was quantified by integration underneath the concentration curves.

#### Hydrogen sulfide and methanethiol

The quadrupole PTR-MS was set at a drift tube pressure, temperature and voltage of 2.15 mbar, 75°C and 600 V, respectively, yielding a reduced electric field number (E/N) of 142 Townsend. The instrument measured hydrogen sulfide isotopologues at nominal *m/z* ratios of 35 and 36 for the [H_2_^32^SH]^+^ and [H_2_^33^SH]^+^ ions. Similarly, methanethiol isotopologues were measured at nominal *m/z* ratios of 49 and 50, for the [CH_4_^32^SH]^+^ and [CH_4_^33^SH]^+^ ions. The humidity dependence of hydrogen sulfide was accounted for by using the calibration procedure described by Feilberg et al. [25]. The proton-transfer-reaction rate constants of methanethiol was taken from Cappellin et al. [26]. The quadrupole PTR-MS dwell time was 200 ms for all relevant *m/z* ratios measured.

The PTR-TOF4000 was run at a drift tube pressure, temperature, and voltage of 2.2–3.3 mbar, 80–119°C, and 600–960 V, respectively, yielding E/N ratios between 152–159 Townsend. The [H_2_^33^SH]^+^ ion and the [NH_4_H_2_O]^+^ cluster ion were measured at *m/z* 35.995 and *m/z* 36.045, respectively. The instrument resolving power, *R*, was ~ 1300–1500 according to *R* = *m*/*Δm*, where *m* is the ion mass at peak maximum and *Δm* is the full width at half maximum of the peak [27]. The PTR-TOF4000 single spectrum time, extraction time, and estimated mass range was 1000 ms, 2000 ns, and 248.3 atomic mass units, respectively.

### Calculation of isotope ratios

The ^13/12^C isotope ratio in methane, carbon dioxide, and acetate is reported using the standard delta notation in Eq 1.

$$\delta^{13}C=\frac{{}^{13/12}C_{\mathrm{sample}}}{{}^{13/12}C_{VPDB}}‐1$$ (1)

where VPDB is the Vienna Peedee belemnite reference, which is defined as 0 ‰ on a scale anchored to consensus values assigned to the reference materials, NBS-19 calcium carbonate (+1.95 ‰) and L-SVEC lithium carbonate (-46.6 ‰) [28]. Consequently, the VPDB ^13/12^C ratio is 0.01118 [29].

The relative methanogenesis pathway contribution was calculated from the quantities, δ^13^C_CH4_ and δ^13^C_CO2_ by fitting measured δ^13^C_CH4_ and δ^13^C_CO2_ to a carbon mass balance model and a known isotope ratio in the methyl carbon of the acetate in the inocula (δ^13^C_2-C-Ac_). The best fit of the model was found by minimizing the difference between measured and modelled δ^13^C_CH4_ and δ^13^C_CO2_ values by adjusting the fraction of methane derived from acetate and the fraction of acetate derived methane from acetoclastic methanogenesis. In the mass balance model it is assumed that methane from acetate is derived from either acetoclastic methanogenesis or syntrophic acetate oxidation coupled to hydrogenotrophic methanogenesis (SAO-HM). For acetoclastic methanogenesis, the carbon in the methyl group of acetate is incorporated into methane and the carbon in the carboxyl group ends up in carbon dioxide. In synthrophic acetate oxidation both carbons goes into two carbon dioxide molecules, before conversion to methane by hydrogenotrophic methanogenesis according to available hydrogen. Consequently, higher δ^13^C_CH4_ ratios can be expected if acetoclastic methanogenesis occurs when the methyl group of acetate is ^13^C-labeled than would be the case for SAO-HM. A detailed description with a full overview of the mass balance model is described in [21].

The δ^13^C_2-C-Ac_ was derived from measurements of acetate concentration in the inocula before addition of known amounts of 2-^13^C-acetate and unlabeled acetate to the inocula. We assumed that the δ^13^C of organic matter in the manure was -28.5 ‰ and that further isotope fractionation associated with fermentative acetate production is negligible [30]. Using these assumptions we assigned a δ^13^C_2-C-Ac_ value of -28.5 ‰ to the unlabeled aceate. These are fair assumptions and often done in isotope signature studies of methanogenic pathways [30]. It is noteworthy to mention that the δ^13^C_2-C-Ac_ is typically slightly lower than the δ^13^C_1-C-Ac_ [30, 31], but this technical nuance is insignificant for the results considering the large pool of labeled acetate in the manures used in this study. Uncertainty in reported δ^13^C_2-C-Ac_ in Table 1 is an artifact of the uncertainty related to the measurement of acetate concentration in the inocula.

The ^33/32^S isotope ratio in sulfate (*R*(^33/32^S)_SO4_), hydrogen sulfide (*R*(^33/32^S)_H2S_), and methanethiol (*R*(^33/32^S)_CH3SH_) is reported as excess *R*(^33/32^S) in units of percentage (%) as no consensus δ^33^S value has been assigned to the Vienna Canyon Diablo Troilite (V-CDT) reference material [32], and because the PTR-MS was not calibrated against a known δ^33^S of a reference material. Instead we used a reference *R*(^33/32^S) value of 0.0078791 as calculated for the IAEA-S-1 reference material by Ding et al. [33]. Excess *R*(^33/32^S) was calculated according to Eq 2.


$$R_{ex}{\left({}^{33/32}S\right)}_{x}=\frac{{}^{33}S_{x}}{{}^{32}S_{x}}-R{\left({}^{33/32}S\right)}_{ref}$$ (2)

Where *x* denotes the sulfur containing compound, *R*_*ex*_ is the excess isotope ratio, and *R*(^33/32^S)_ref_ is the reference *R*(^33/32^S) equal to 0.0078791. The *R*_*ex*_(^33/32^S)_SO4_ in the swine manure was calculated based on sulfate concentrations measured in the swine manure before addition of known amounts of sodium sulfate and sodium ^33^S-sulfate.

Hydrogen sulfide isotopologues were measured at *m/z* ratios of 35 and 36 and methanethiol isotopologus were measured at *m/z* ratios of 49 and 50. Contribution of ^13^C to measured *m/z* ratios were subtracted by assuming an *R*(^13/12^C) of 1.086 (%), which is equivalent to a δ^13^C value of -28.5 ‰. Uncertainty in reported *R*_*ex*_(^33/32^S)_SO4_ in Table 1 is derived from the uncertainty of the sulfate concentration measurement in the unlabeled inocula.

### Inoculum characterization

The volatile fatty acids content was analyzed on a HP 6850 Series GC system (Agilent Technologies, Santa Clara, CA, USA) using the sample preparation described by Mulat et al. [34]. Sulfate concentrations in the manure (prediluted 50–2000 times) were analyzed with a Sulfate Test Kit 1.01812.0001 (Merck) on a Spectroquant NOVA 60 (Merck). Total ammonia nitrogen was measured with a Spectroquant NOVA 60 (Merck) using an ammonium test kit 1.00683 (Merck) or measured by the standard Nessler method using a DR 3900 benchtop spectrometer (Hach-Lange, Loveland, CO, USA) [35]. Total solids and volatile solids content were analyzed gravimetrically by the standard method [36] by heating 20 g manure at 105°C for 24 h and subsequently burning it at 550 for 6 h in a furnace oven (Nabertherm).

### Microbial community structure

Two different approaches were used to study microbial community structure. For methanogens the alpha subunit of the methyl coenzyme M reductase was analyzed with the terminal restriction fragment length polymorphism method. This method is a robust fingerprint technique and a legitimate approach for analyzing archaeal taxonomy down to the family level [37, 38]. For sulfate reducers we targeted the domain-specific 16S rRNA gene, which is ubiquitous in all bacteria, and have been targeted specifically to study sulfate reducer populations [39–41].

#### DNA extraction

Thawed manure samples (400–500 μL) were used to extract DNA using a NucleoSpin soil kit (Macherey-Nagel GmbH & Co. KG, Düren, Germany). The DNA quality was checked with a 0.8% agarose gel electrophoresis and the concentration was determined using a NanoDrop ND-1000 UV/visible spectral photometer (PeqLab, Germany) and a Qubit dsDNA BR Assay kit (Invitrogen, Waltham Massachusetts, USA).

#### Terminal restriction fragment length polymorphism

The alpha subunit of the methyl coenzyme M reductase (*mcrA*) gene was polymerase chain reaction (PCR) amplified using the mlas and mcrA-rev primer set and PCR program described earlier [42], which produces PCR products of approximately 500 bp. The mcrA-rev primer was 5’-labeled with the phosphoramidite fluorochrome, 6-carboxyfluorescein (FAM). The PCR products were purified using a SureClean plus kit (Bioline, Germany). The quality was checked by gel-electrophoresis using a 1.5% agarose gel and quantified with a NanoDrop ND-1000 UV. Following purification, the PCR products (10–30 ng per sample) were digested with BstNI (2 hours incubation at 60°C) and MwoI (37°C overnight) restriction enzymes. The digested PCR products were precipitated with 30 μL absolute ethanol and 2.5 μL EDTA, and the centrifuged pellet was washed in 70% ethanol. The washed and dried digested PCR products were resuspended in HiDi formamide with the fragment size standard GeneScan-500 ROX (Applied Biosystems GmbH, Weiterstadt, Germany). The fluorescently labeled terminal restriction fragments were separated via capillary electrophoresis with an automatic sequencer (ABI Prism 3130xl genetic analyzer; Applied Bio-systems, Weiterstadt, Germany). The taxonomy was assigned to the terminal restriction fragments using the database described in [43].

### 16S rRNA gene analysis

Sulfate reducing bacteria community structure was assessed by PCR amplifying the V3-V4 variable regions of the bacterial 16S rRNA gene using the 341f (5’-CCTACGGGNGGCWGCAG-3’) and 785r (5’-GACTACHVGGGTATCTAATCC-3’) primer set [44]. The PCR products were purified with Agencourt AMPure XP magnetic beads and a magnetic stand (Beckman Coulter, Brea, California, USA). An index PCR on the purified PCR products was carried out using a Nextera XT DNA Library Preparation Kit (Illumina, San Diego, Californien, USA). The cleaned index PCR products were diluted and sequenced with the Illumina MiSeq amplicon sequencer (Illumina V3, 2X300bp). The raw sequencing data was processed in QIIME2 bioinformatics platform 2018.11 [45]. Denoising of paired-end reads, dereplication, chimera filtering, and generation of Amplicon Sequence Variants (ASVs) were made with the DADA2 plugin [46]. The taxonomy was assigned to the ASVs using the MiDAS 2.1.3 reference database built for the V3 –V4 hypervariable regions, respectively [47]. The ASV frequency table, taxonomy, and DNA sequences were exported from QIIME2 objects to text and FASTA files for data analysis. The sequences obtained from this study were deposited in the NCBI SRA public database under the accession numbers SRR12046941—SRR12046973.

### Statistics

Statistical significance of gas measurements was tested with one-way ANOVA with a level of significance (α) of 0.05 using Microsoft Excel (2016). After one-way ANOVA a Tukey Kramer post hoc test was performed and the pairwise comparison between groups was evaluated based on the q statistic using α = 0.05. For microbial community analysis the non-metric multidimensional scaling (NMDS) plots were made in R [48] using the vegan package and the “envfit” function. Uncertainties reported in the main text are 95% confidence intervals unless stated otherwise.

## Results

This section describes results from the main experiments that is incubations with swine manure treated with TA-NaF or acidification. Results of supporting experiments are mentioned briefly in the relevant context, but for further details see S1 and S2 Appendices. Characteristics of all studied inocula are presented in S4 Appendix.

### Methanogenesis pathways–Experiment 1

**Fig 3:**
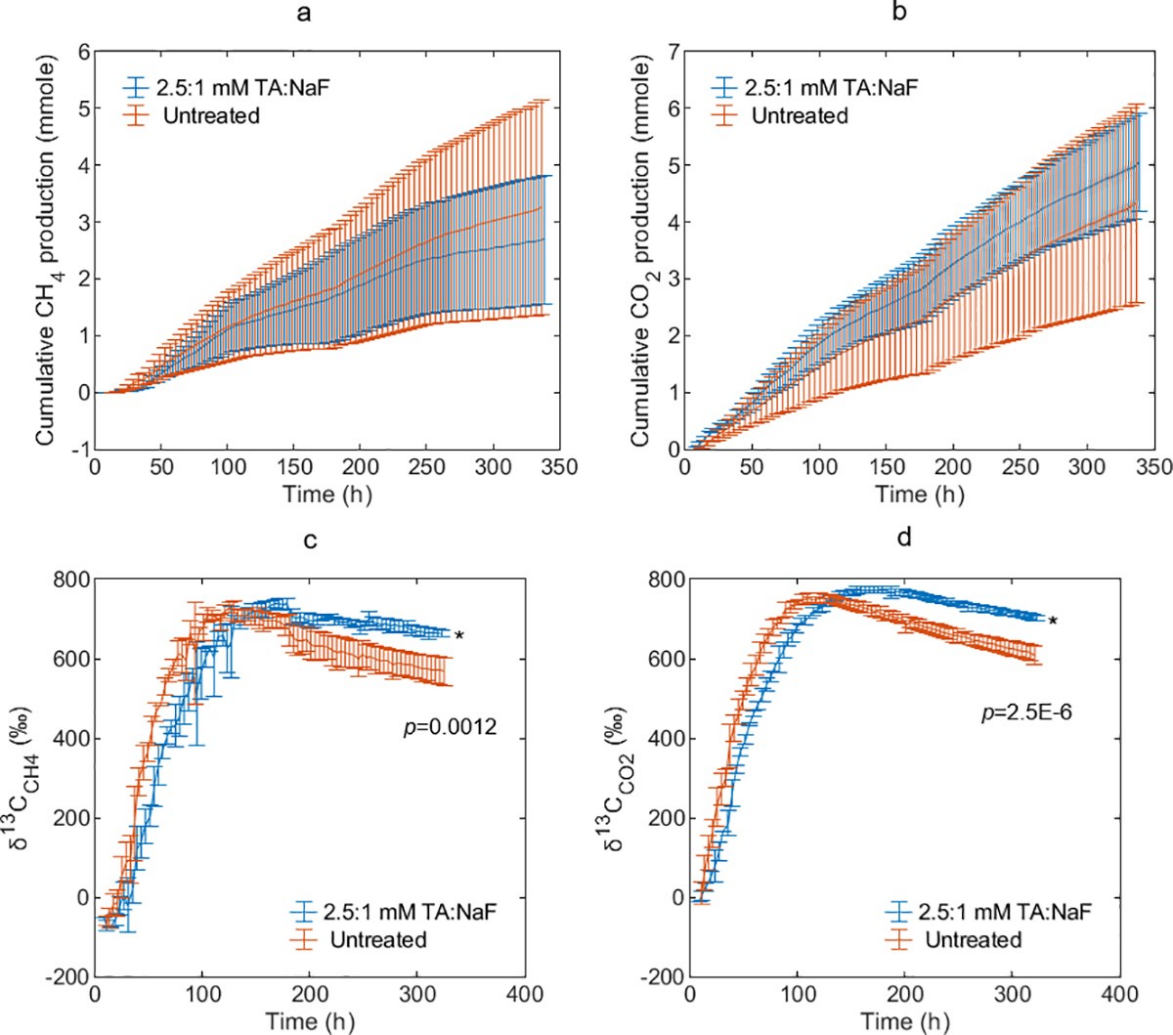
Production of methane (a) and carbon dioxide (b). Isotope ratios of methane (c) and carbon dioxide (d). TA:NaF is tannic acid to sodium fluoride ratio. Data is presented as the mean ± SD of triplicates. Curves with different number of * are different by experiment end according to one-way-ANOVA. p-value is for one-way-ANOVA.

Fig 3 shows δ^13^C_CH4_, δ^13^C_CO2_, methane, and carbon dioxide production from 2-^13^C-acetate labeled swine manure amended with TA-NaF or left untreated. The δ^13^C_2-C-Ac_ in the swine manures was estimated to 7719 ± 451 ‰. Cumulative methane emission was slightly lower in TA-NaF treated manure than in untreated manure, but the reduction was not statistically significant. This is in agreement with Dalby et al. [17], where no difference was found in methane production during the first 14 days of incubation with 2.5:1 mM TA:NaF. Carbon dioxide production in TA-NaF treated swine manure was not significantly different from untreated swine manure either (Fig 3B). In Fig 3C and 3D, δ^13^C_CH4_ and δ^13^C_CO2_ values were lower in TA-NaF treated manures during the initial 4–5 days but higher for rest of the experiment. This stresses an important point–that methanogenesis was affected by TA-NaF treatment, even though it was not clearly reflected in the cumulative gas production. δ^13^C_CH4_ and δ^13^C_CO2_ values were very similar in absolute values and in temporal evolution irrespective of treatment, which suggest that syntrophic acetate oxidation coupled to hydrogenotrophic methanogenesis (SAO-HM) was dominating in both TA-NaF treated and untreated swine manure. This was concluded since pure acetoclastic methanogenesis from a stoichiometric perspective will only increase δ^13^C_CH4_. By fitting the carbon mass balance model with measured isotope ratios it was estimated that more than 99% of the acetate derived methane came from SAO-HM, and 21% of the total methane was derived from acetate at peak δ^13^C_CH4_ values. Untreated swine manure that was not amended with labeled acetate suggested likewise that the swine manure was dominated by hydrogenotrophic methanogens having δ^13^C_CH4_ values of -79.9 ± 0.9 ‰ in the start and -67.4 ± 4.4 ‰ by experiment end, and δ^13^C_CO2_ values 7.8 ± 2.1 ‰ in the start and 10.5 ± 4.6 ‰ by experiment end. Such large differences between methane and carbon dioxide isotope signatures are typically observed when methanogenesis mainly occurs via the hydrogenotrophic pathway [30].

Consequently, the experiments were inconclusive to whether acetoclastic methanogenesis was inhibited by TA-NaF treatment. We therefore applied the same isotope labeling approach to cattle manure and wastewater sludge, hoping that the acetoclastic methanogenesis was more pronounced in the untreated inocula. In wastewater sludge, isotope ratios clearly indicated that the untreated inocula as dominated by acetoclastic methanogenesis. With TA-NaF treatment a switch in δ^13^C values were observed, that clearly indicated inhibition of acetoclastic methanogenesis. Acidification with hydrochloric acid inhibited, on the other hand, SAO-HM. The TA-NaF treated and untreated cattle manures were almost exclusively dominated by SAO-HM. Cattle manure, which was acidified with sulfuric acid showed very high δ^13^C_CO2_ values, which were inexplicable if accounting solely for methanogenesis processes. Instead, this suggested that sulfate reducing bacteria, rather than methanogens, consumed the 2-^13^C-acetate. The detailed description of the method and results are found in S1 and S2 Appendices.

### Reduced sulfur compounds production–Experiment 2

**Fig 4:**
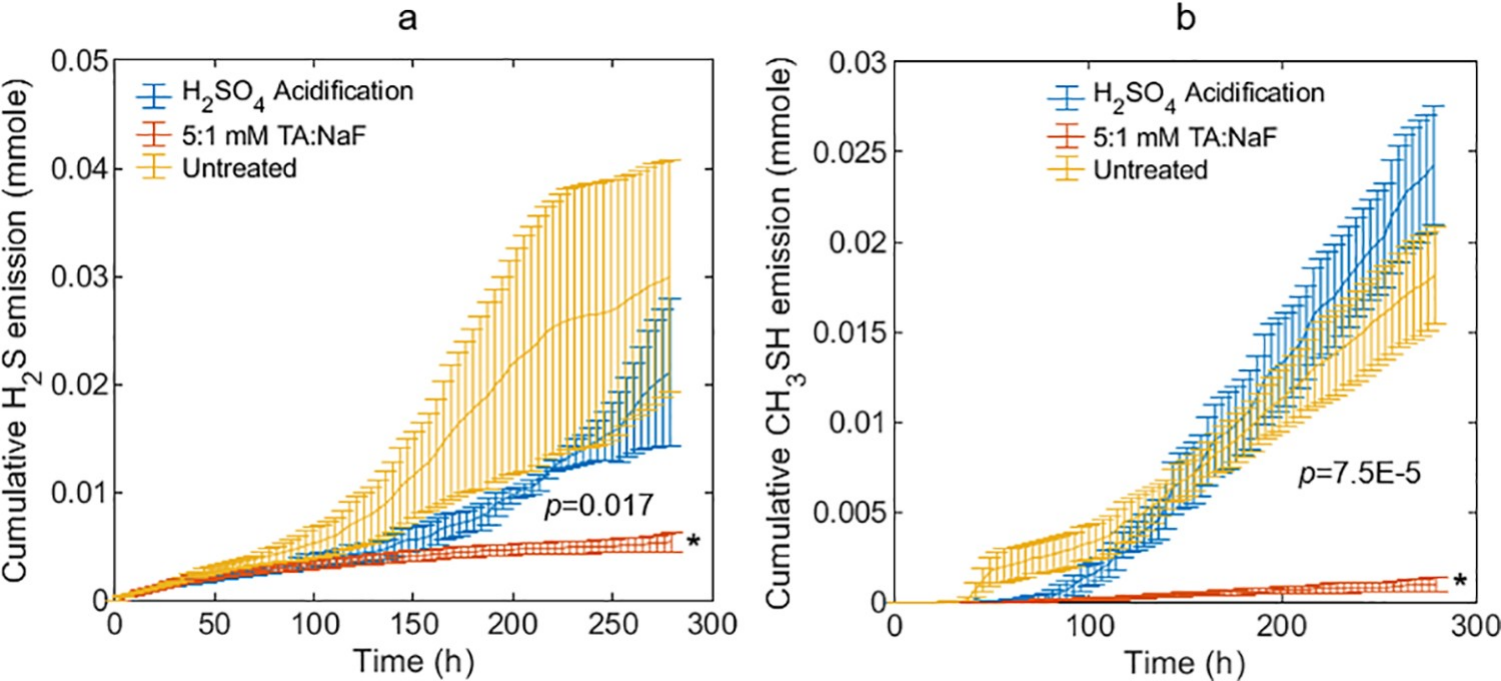
(a) Hydrogen sulfide (H_2_S) emission and (b) methanethiol (CH_3_SH) emission from fresh swine manure treated with TA-NaF or acidified with sulfuric acid to pH 5.5. Data is presented as the mean ± SD of triplicates. Curves with different number of * are different by experiment end according to one-way-ANOVA. p-value is for one-way-ANOVA.

We conducted headspace experiments on fresh swine manure while measuring hydrogen sulfide and methanethiol emissions. Fig 4A shows that TA-NaF treatment reduced hydrogen sulfide emission significantly by 82.1 ± 7.0%, whereas a smaller and not statistically significant reduction of 29.2 ± 34% was observed for acidification (H_2_SO_4_) treatment to pH 5.5. Hydrogen sulfide emission was also reduced upon acidification in similar studies on swine and cattle manure [22, 49], which supports our findings. In Fig 4B methanethiol emission was reduced significantly by 94.5 ± 2.5% with TA-NaF treatment. On the contrary, acidification increased methanethiol emissions by 33.8 ± 27.6%, which is consistent with other studies [49, 50].

### Reduced sulfur compounds pathways–Experiment 3

**Fig 5:**
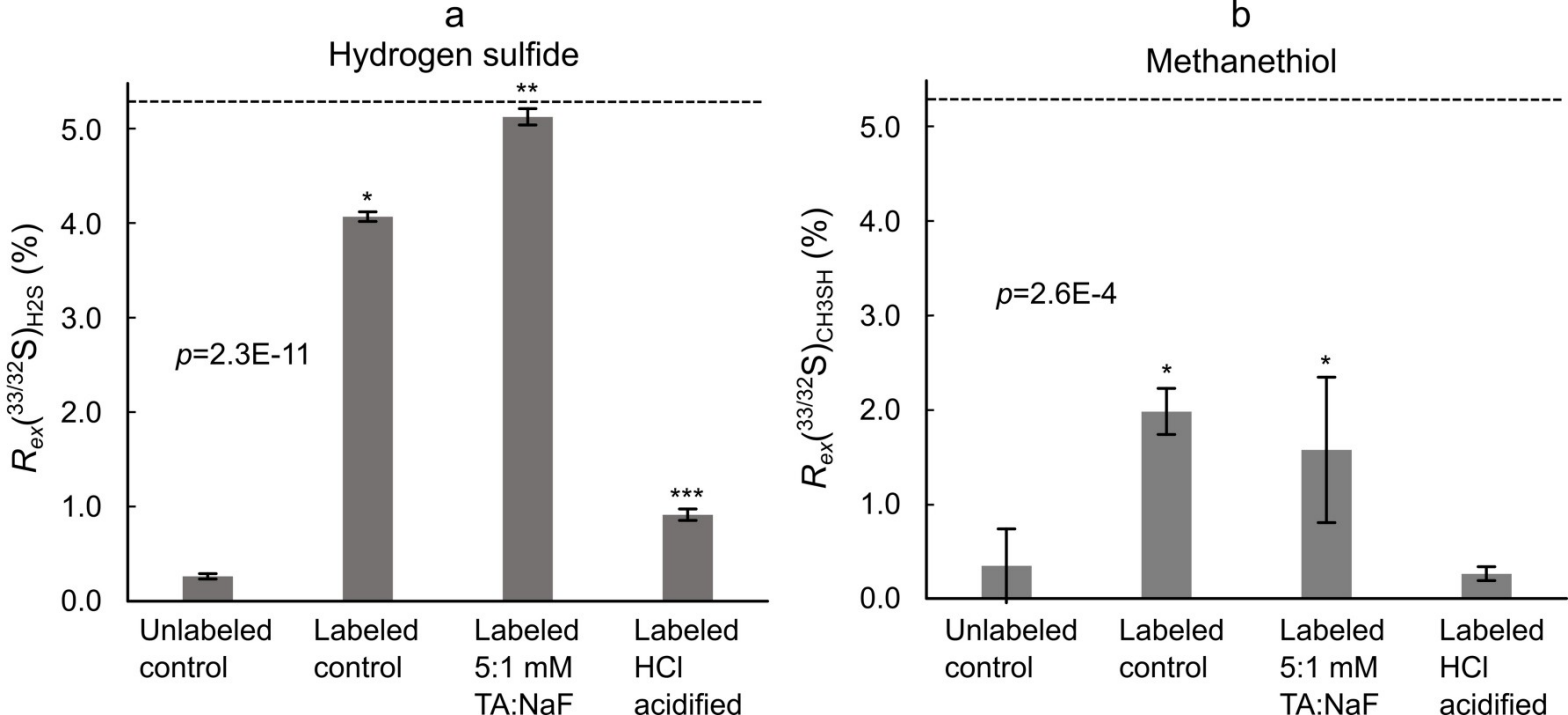
Isotopologue ratios of (a) hydrogen sulfide (H_2_S) and (b) methanethiol (CH_3_SH) from ^33^S-sulfate labeled swine manure treated with TA-NaF or acidified with hydrochloric acid (HCl). The dashed line is the *R*_*ex*_(^33/32^S)_SO4_. Data is presented as the mean ± 95% confidence interval. Bars with different number of * are different according to one-way-ANOVA. p-value is for one-way-ANOVA test. The data used is averaged over 2 hours and was measured at day 4.

The *R*_*ex*_(^33/32^S)_SO4_ was estimated to be 5.28 ± 0.53% based on the amount of unlabeled sulfate present in the inoculum and the amount of ^33^S-sulfate added. Potential interference at the [H_3_^33^S]^+^ ion on the PTR-MS was reduced by applying a Nafion tube as described in S3 Appendix. Fig 5 presents isotopologue ratios of hydrogen sulfide and methanethiol from the ^33^S-sulfate labeled swine manure bottles after four days of incubation in the headspace gas monitoring setup (Fig 2). The isotope ratio in unlabeled control manure was in close agreement with the theoretical isotope ratio based on a reference *R*(^33/32^S) of 0.788% [33]. The labeled controls indicate that approximately 80% of the hydrogen sulfide was produced by reduction of sulfate (Fig 5A), which is consistent with previous estimates of 77 ± 3% in swine manure [7]. Treatment with TA-NaF yielded a *R*_*ex*_(^33/32^S)_H2S_ value similar to the *R*_*ex*_(^33/32^S)_SO4_ value, suggesting that more than 95% of the emitted hydrogen sulfide was derived from sulfate reduction. This implies that cysteine catabolism was severely inhibited upon TA-NaF treatment. At the same time Fig 4A shows that sulfate reduction must also have been partially inhibited by TA-NaF to account for the large reduction in hydrogen sulfide emission. Contrarily, acidification resulted in relatively low *R*_*ex*_(^33/32^S)_H2S_ values, which indicated that sulfate reduction was inhibited relatively more than cysteine degradation. Fig 5B shows that acidification resulted in a low *R*_*ex*_(^33/32^S)_CH3SH_ values, resembling the isotope ratio to be expected in unlabeled methanethiol. The *R*_*ex*_(^33/32^S)_CH3SH_ was lower than in *R*_*ex*_(^33/32^S)_H2S_, which could be an effect of inhibited hydrogen sulfide methylation combined with a relatively higher contribution from methionine. We observed no significant difference between TA-NaF treatment and labeled control reactors at *m/z* 50: *m/z* 49. However, the difference between *R*_*ex*_(^33/32^S)_H2S_ (Fig 5A) and *R*_*ex*_(^33/32^S)_CH3SH_ (Fig 5B) was relatively larger for TA-NaF treated manure (3.57 ± 0.78%) than for the untreated control manure (2.10 ± 0.25%). It is thus likely that TA-NaF exhibits a pronounced inhibition effect on hydrogen sulfide methylation.

### Microbial community structure–Experiment 4

#### Methanogens

**Fig 6:**
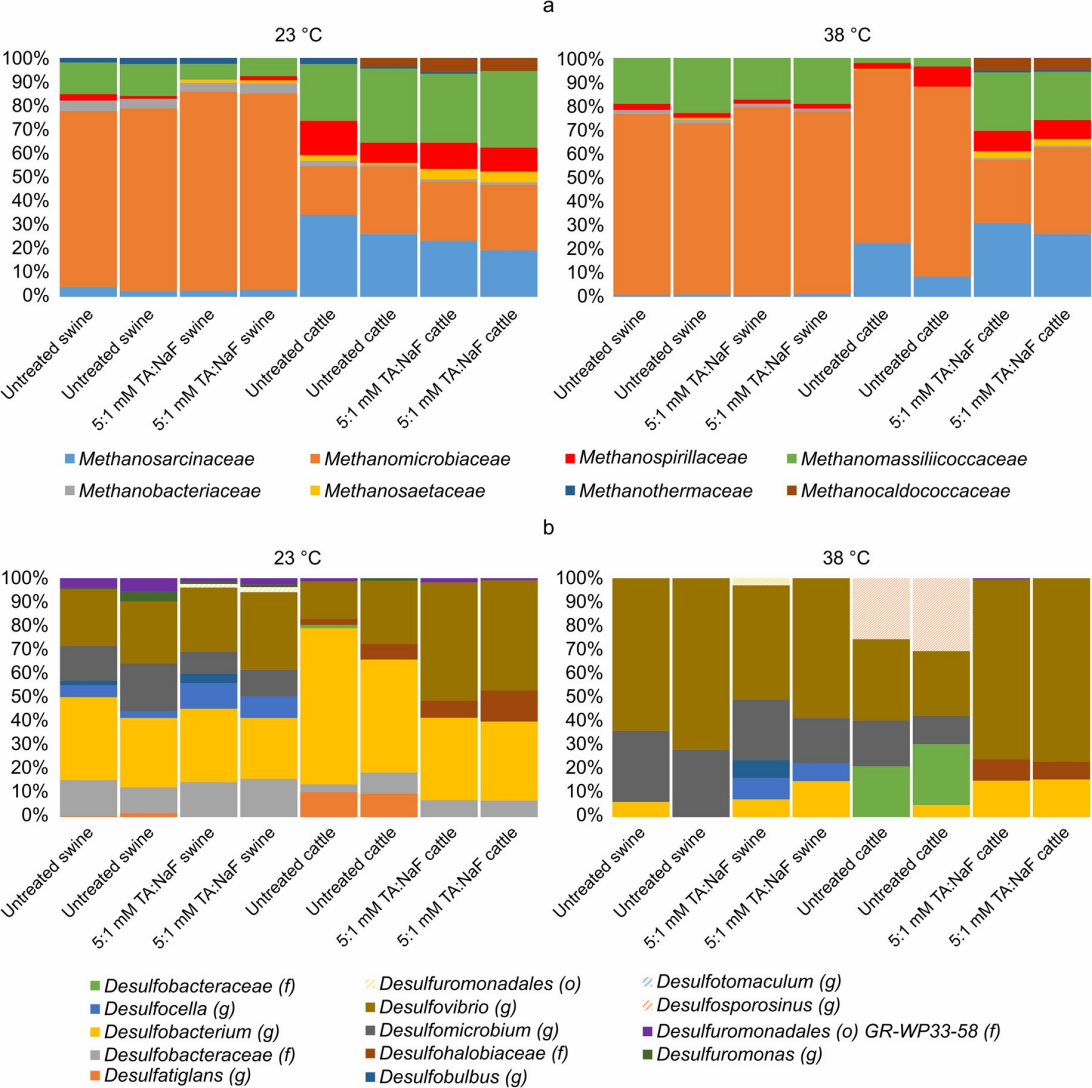
Relative abundance of methanogens at a family level in untreated and TA:NaF treated swine and cattle manure (a). Relative abundance of sulfate reducing bacteria (b), with taxonomic levels specified with (g) for genus, (f) for family, and (o) for order.

Fig 6A shows the relative abundance of methanogens at a family level for untreated and TA-NaF treated swine and cattle manure. *Methanomicrobiaceae* was abundant in swine manure at both 23°C and 38°C and in cattle manure at 38°C. On the other hand, *Methanosarcinaceae* was almost absent in swine manure, but well represented in cattle manure. Untreated cattle manure at 38°C seemed to be dominated more by *Methanomicrobiaceae*. This dominance was not seen for TA-NaF treated cattle manure at 38°C where the community structure resembled that in cattle manure incubated at 23°C, suggesting that TA-NaF inhibited the growth of *Methanomicrobiaceae*.

**Fig 7:**
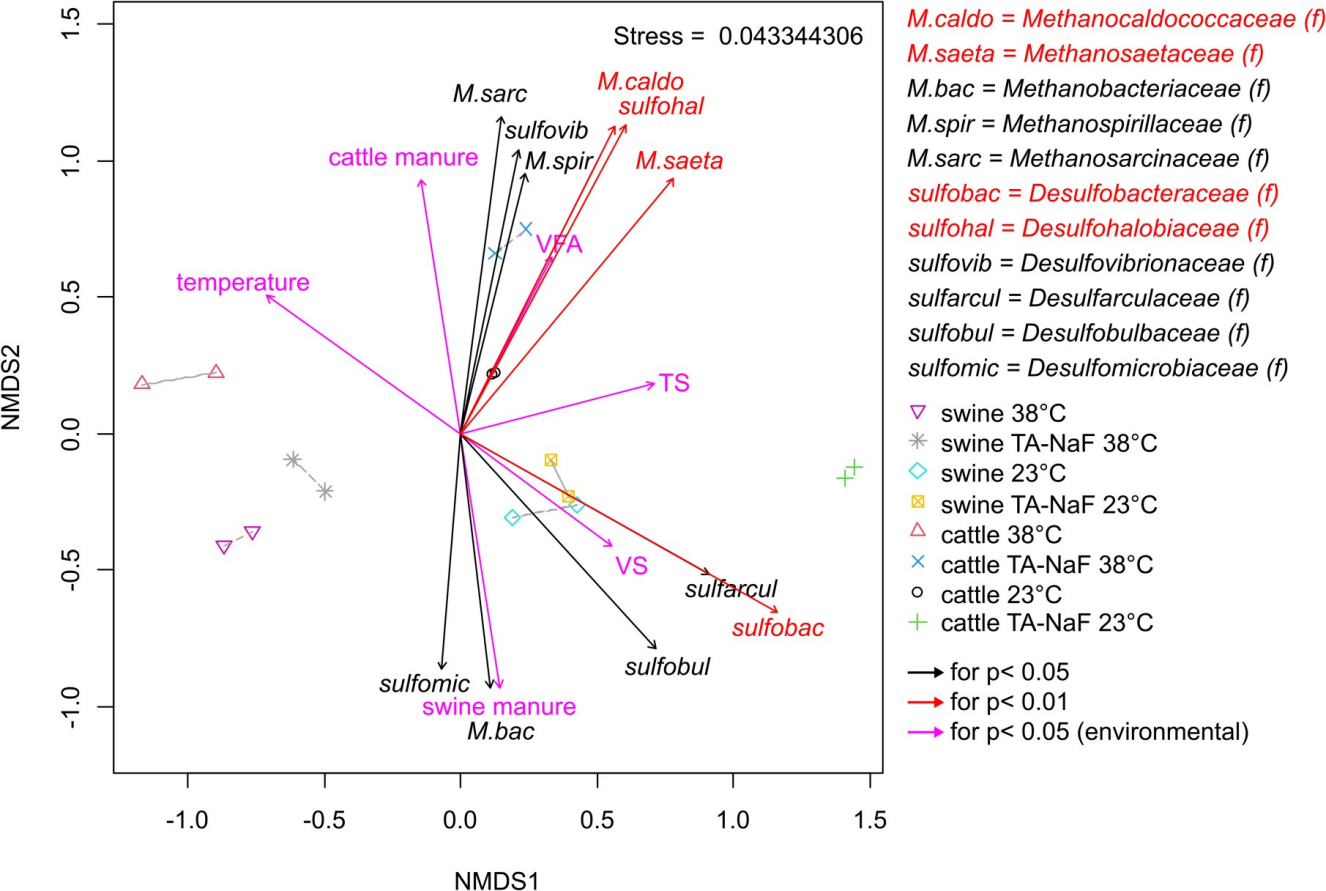
Non-metric multidimensional scaling of microbial community analysis and environmental parameters after 30 days of manure incubation. (f) is family taxonomy level.

Fig *7* shows a Non-metric multidimensional scaling plot. TA-NaF treatment had no significant effect on methanogenic community structure after 30 days. There were no statistically significant correlations between TA-NaF treatment and the methanogenic community structure in swine or cattle manure. However, Methanosarcinaceae was correlated with cattle manure whereas Methanobacteriaceae was correlated with swine manure. There were no clear correlations between temperature and methanogen community structure.

#### Sulfate reducing bacteria

Sulfate reducing bacteria were detected only within the *Deltaproteobacteria* and *Clostridia* classes, where most sulfate reducing bacteria genera belong [51]. The relative abundance of sulfate reducing bacteria are presented in Fig 6B. In general, *Desulfovibrio* was more dominant at 38°C and *Desulfobacterium* was more dominant at 23°C for both swine and cattle manures. *Desulfocella* in swine manure and the *Desulfohalobiacea* family in cattle manure adapted well to TA-NaF treatment, irrespective of incubation temperature. On the contrary, *Desulfatiglans* was inhibited in both manure types at 23°C. The same genera adapted well at 38°C, where particularly *Desulfobacteraceae*, *Desulfosporosinus*, and *Desulfomicrobum* were outcompeted in cattle manure. TA-NaF treatment had no statistically significant effect on the community structure of sulfate reducing bacteria in the NMDS plot in Fig 7. Manure type was more important and *Desulfomicrobiaceae* was correlated with swine manure, but for cattle manure there were correlations with *Desulfohalobiaceae* and *Desulfovibrionaceae*.

## Discussion

### Methanogenesis pathways

The effect of TA-NaF in swine manure was not very pronounced for the production of methane and carbon dioxide. This could be associated with the lower treatment dose (2.5:1 mM) compared to previous studies [17, 18] where 5:1 or 10:1 mM were used. A low treatment dose was, however, chosen to ensure that severe inhibition would not limit gas production to the extent where isotope ratios could not be measured. In addition, the volatile solids content of the inocula had to be considered as the TA-NaF inhibition effect is reduced in inoculum with higher organic content, as tannic acid may precipitate with proteins rather than binding to the cell membranes of methanogens and bacteria [17]. The dominance of hydrogenotrophic methanogens in swine manure is consistent with earlier measurements of the isotope signatures of methane with gas chromatography combustion isotope ratio mass spectrometry [18]. In that study, isotope signatures from TA-NaF inhibited swine manure were not different from the signatures of untreated manure. To avoid such inconclusive results, it is necessary to use inocula with a balanced methanogenic community structure, where both methanogenesis pathways are equally active. For that reason wastewater sludge, which is often dominated by acetoclastic methanogens [52, 53], and cattle manure was also examined in this study.

The methane reduction in sulfuric acid acidified cattle manure is consistent with several studies on acidification effects on gas emissions [22, 54, 55]. The acidic environment, which can cause volatile fatty acid uncoupling effects inside cells [56], might partially explain methane reduction upon acidification treatment. However, in wastewater sludge acidified by hydrochloric acid, reductions in methane emission were not observed, which indicates that sulfate, derived from sulfuric acid, plays a possible role in reducing methane production. This deduction was supported by the extremely high ẟ^13^C_CO2_ values from acidified cattle manure, indicating that sulfate reducers, rather than methanogens, were consuming the ^13^C-labeled acetate. Furthermore, ẟ^13^C_CO2_ values were very low in hydrochloric acid acidified wastewater sludge in which the sulfate content was orders of magnitude lower.

When the sulfate content is high, sulfate reducing bacteria may outperform methanogens [51] or even inhibit methanogenesis due to sulfide production [57, 58]. The chemical oxygen demand/sulfate ratio has been reported to be an important factor ultimately determining whether sulfate reducers or methanogens dominate, but the critical value where the dominance tip occurs vary considerably in the literature [59]. Molybdate is an inhibitor of sulfate reduction [60, 61] and it could be applied to rule out and quantify the importance of acetate consumption by sulfate reduction.

### Reduced sulfur compounds

In TA-NaF treated swine manure, production of reduced sulfur compounds was severely reduced. Though cysteine degradation was almost completely inhibited, it remains unclear exactly how methionine degradation was affected. It has been shown that methanethiol from swine manure originates primarily from methionine degradation [7], and hence a huge reduction in methanethiol production, as those seen for TA-NaF treatment in this study, is likely to result from inhibition of methionine degradation. The limited production of methanethiol made it challenging to achieve the precision needed to differentiate between the untreated control manure and TA-NaF treated manure. A modified approach, which accounts for the low methanethiol emission from TA-NaF treated manure, should be developed in order to make more accurate assessments of the effect on methanethiol formation. This might be realized by reducing the inhibitor dose or by concurrent TA-NaF and methionine supplementation. Hydrochloric acidification increased methanethiol emission slightly and reduced sulfate reduction activity in swine manure. This is in agreement with other studies using sulfuric acid acidification [17, 22, 49, 50].

### Microbial community structure

In general, there was no significant changes in methanogen community upon TA-NaF treated manure at 23°C compared to untreated manure, which was surprising considering the large influence TA-NaF at a 5:1 mM dose had on methane production in another study [18]. This could suggest that TA-NaF inactivates metabolic activity but is not lethal [17] or that the DNA of dead cells was still present when PCR analysis was conducted. This testifies to the fact that the TA-NaF treatment effects are complex and our understanding of its influence on a cellular level is yet very limited. The dominant sulfate reducers in TA-NaF treated manure was halophile sulfate reducing bacteria, which are found in hyper-saline environments [62]. This discovery suggests that TA-NaF induces cellular stress in a similar manner to high salt concentrations. Whitehead et al. [63] previously examined the sulfate reducer community in pig slurry and found that particularly *Desulfobulbus* was sensitive to quebracho tannin treatment. However, in our experiments, *Desulfobulbus* was more abundant in TA-NaF treated swine manure than in the control manure. Here it is noteworthy to mention that Whitehead et al. used quebracho tannin [63], which is fundamentally different in chemical structure to TA and their results were based on sequencing of the subunit A of the dissimilatory sulfite reductase gene (*dsrA*).

### Effect on biological transformations

**Fig 8:**
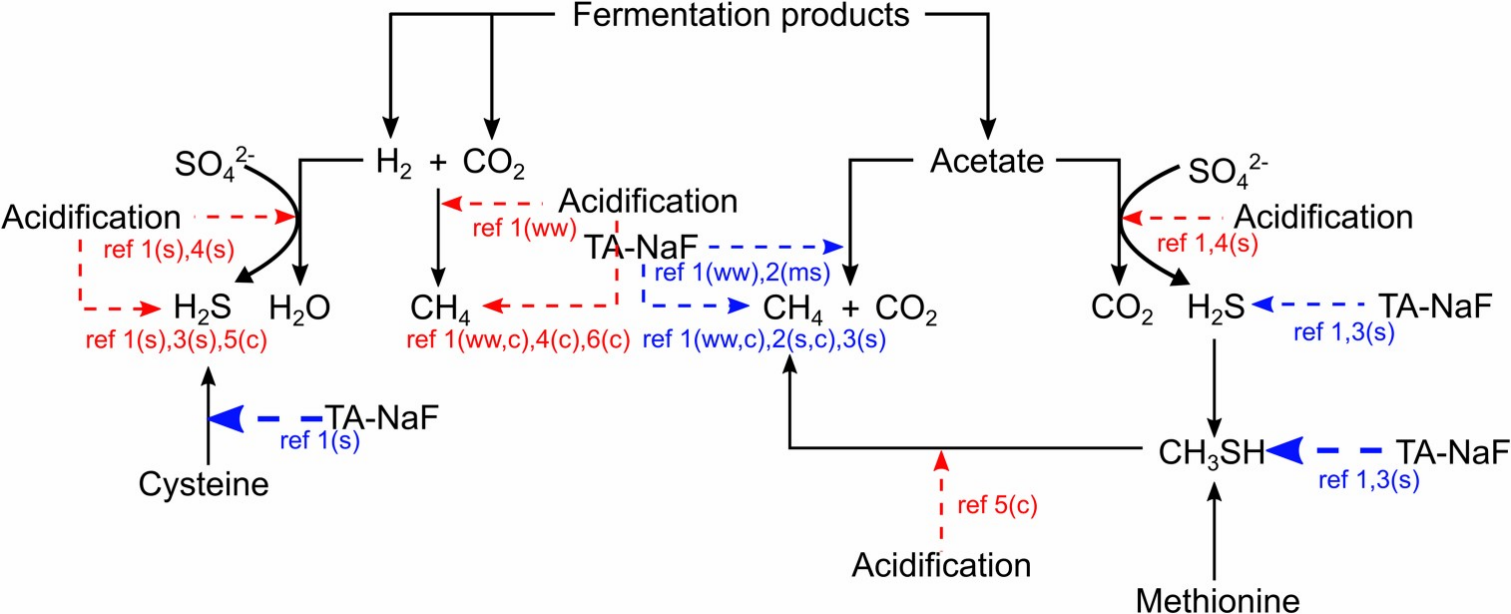
Inhibition (dashed arrows) of sulfur transformations and methanogenesis by TA-NaF (tannic acid with sodium fluoride) or acidification treatment of swine manure and other inocula. Blue arrows and text indicate inhibition effect seen for TA-NaF treatment. Red arrows and text indicate inhibition effect seen for acidification treatment. Thicker arrows indicate stronger inhibition than thin arrows of the same color. When inhibition arrows point at compounds, it indicates that emission of that compound was reduced but the inhibited pathway was not identified. Ref 1 is this study, ref 2 is Dalby et al. 2020, Bioresour. Technol, ref 3 is Dalby et al. 2020, Environ. Sci. Technol., ref 4 is Ottosen et al. 2009, ref 5 is Eriksen et al. 2012, ref 6 is Petersen et al. 2012. For the references, (s) indicates swine manure was used, (c) indicates cattle manure, (ww) indicates wasterwater sludge, and (ms) indicates maize sillage.

The foregoing knowledge combined with previous fundamental studies on acidification [22, 49, 54] and TA-NaF effects [17, 18] on sulfur transformations and methanogenic pathways in swine manure led to the proposed inhibition scheme in Fig 8. Fig 8 portrays inhibition mechanisms observed mostly for swine manure but may also be valid for cattle manure, wastewater sludge, and maize silage. It is plausible that different inocula and methanogenic environments will lead to different inhibition mechanisms. For example, acidification can lead to both acetoclastic and hydrogenotrophic dominance depending on the rate of change in pH in sludge flocs [64]. It also remains unclear whether acetoclastic, hydrogenotrophic, or sulfate reducing bacteria utilizing numerous fermentation products as electron donors [10, 51] are affected to differing degrees by either treatment method. Ultimately, a more comprehensive labeling strategy involving simultaneous ^13^C-substrate and ^33^S-sulfate could shed more light on this matter.

Since acidification does not reduce methanethiol emission, the combination of TA-NaF treatment with acidification is an attractive idea, as these additives seems to inhibit all the critical pathways in production of methane and reduced sulfur compounds (Fig 8). A natural extension of the presented work should therefore include test of combined TA-NaF and acidification treatment but also large scale testing in e.g. livestock buildings or slurry storage containers.

## Supporting information

S1 Appendix Methanogenesis in cattle manure. Production of methane (a), emission of carbon dioxide (b), δ^13^C_CH4_ values (c), and δ^13^C_CO2_ values (d-e) from degassed cattle manure treated with tannic acid and fluoride (TA-NaF) or acidified with sulfuric acid to around pH 5.5.
(DOCX)

S2 Appendix Methanogenesis in wastewater sludge. Production of methane (a), emission carbon dioxide (b) from acidified and TA-NaF treated wastewater sludge. δ^13^C_CH4_ values (c) and δ^13^C_CH4_ values (d).
(DOCX)

S3 Appendix PTR-MS interference. (a) NH_4_H_2_O^+^ cluster ions with and without a Nafion tube prior to the PTR-MS inlet from swine manure emitting ammonia at concentrations between 30 and 40 ppm (measured at a reduced electric field of 159 Townsend). In the upper right corner of (a) spectral overlap between NH_4_H_2_O^+^ and H_2_^33^SH^+^ ions is observed at 5 ppm ammonia and 4–5 ppm hydrogen sulfide (measured at a reduced electric field of 152 Townsend). “Nafion” and “Background” in (a) indicate at what time a Nafion tube and a charcoal filter (reflecting background signal) were applied in the measurement setup, respectively. (b) Measured ion ratios corresponding to the m/z ratios were hydrogen sulfide and methanthiol isotopologues are measured (Nafion tube applied). Full black lines in (b) indicate theoretical m/z ratios of the [H_3_S]^+^ and [CH_4_SH]^+^ ions (measured at a reduced electric field of 152 Townsend).
(DOCX)

S4 Appendix Manure characterization. Chemical characterization of the inocula used in this study. Uncertainty is presented as the standard deviation of triplicate samples. N/A denotes that the quantity was not measured. TS = Total solids, VS = Volatile solids, TAN = Total ammonia nitrogen, TN = Total nitrogen, and VFA = Volatile fatty acids.
(DOCX)

## References

[1] Ali Shah F, Mahmood Q, Maroof Shah M, Pervez A, Ahmad Asad S. Microbial ecology of anaerobic digesters: The key players of anaerobiosis. Sci World J. 2014;2014. doi: 10.1155/2014/183752 24701142 PMC3950365

[2] Tram V.O. P, Ngo HH, Guo W, Zhou JL, Nguyen PD, Listowski A, et al. A mini-review on the impacts of climate change on wastewater reclamation and reuse. Sci Total Environ. 2014;494–495: 9–17. doi: 10.1016/j.scitotenv.2014.09.107 25020098

[3] Angelidaki I, Karakashev D, Batstone DJ, Plugge CM. Stams, Alfons J M. Biomethanation and its potential. 1st ed. Methods Enzymol. Elsevier Inc.; 2011. doi: 10.1016/b978-0-12-385112-3.00016–0 21402222

[4] Holmes DE, Smith JA. Biologically produced methane as a renewable energy source. Adv Appl Microbiol. 2016;97: 1–61. doi: 10.1016/bs.aambs.2016.09.001 27926429

[5] Feilberg A, Hansen MJ, Liu D, Nyord T. Contribution of livestock H2S to total sulfur emissions in a region with intensive animal production. Nat Commun. 2017;8: 1–7. doi: 10.1038/s41467-016-0009-6 29051487 PMC5648877

[6] Dias B, Weimer B. Conversion of methionine to thiols by lactococci, lactobacilli, and brevibacteria. Appl Environ Microbiol. 1998;64: 3320–3326. doi: 10.1128/AEM.64.9.3320-3326.1998 9726877 PMC106727

[7] Dalby FR, Hansen MJ, Feilberg A. Application of Proton-Transfer-Reaction Mass Spectrometry (PTR-MS) and 33S Isotope Labeling for Monitoring Sulfur Processes in Livestock Waste. Environ Sci Technol. 2018;52: 2100–2107. doi: 10.1021/acs.est.7b04570 29338206

[8] Higgins MJ, Chen Y-C, Yarosz DP, Murthy SN, Maas NA, Glindemann D, et al. Cycling of Volatile Organic Sulfur Compounds in Anaerobically Digested Biosolids and its Implications for Odors. Water Environ Res. 2006;78: 243–252. doi: 10.2175/106143005x90065 16629264

[9] Feilberg Tavs Nyord A, Hansen MN, Lindholst S. Chemical Evaluation of Odor Reduction by Soil Injection of Animal Manure. J Environ Qual. 2011;40: 1674. doi: 10.2134/jeq2010.0499 21869529

[10] Liamleam W, Annachhatre AP. Electron donors for biological sulfate reduction. Biotechnol Adv. 2007;25: 452–463. doi: 10.1016/j.biotechadv.2007.05.002 17572039

[11] Thauer RK, Jungermann K, Decker K. Energy conservation in chemotrophic anaerobic bacteria. Bacteriol Rev. 1977;41: 100–180. doi: 10.1128/br.41.1.100-180.1977 860983 PMC413997

[12] Jin Q, Kirk MF. Thermodynamic and kinetic response of microbial reactions to high CO 2. Front Microbiol. 2016;7. doi: 10.3389/fmicb.2016.01696 27909425 PMC5112241

[13] Hattori S. Syntrophic acetate-oxidizing microbes in methanogenic environments. Microbes Environ. 2008;23: 118–127. doi: 10.1264/jsme2.23.118 21558697

[14] Schink B. Energetics of syntrophic cooperation in methanogenic degradation. Microbiol Mol Biol Rev. 1997;61: 262–280. doi: 10.1128/mmbr.61.2.262-280.1997 9184013 PMC232610

[15] Westerholm M, Moestedt J, Schnürer A. Biogas production through syntrophic acetate oxidation and deliberate operating strategies for improved digester performance. Applied Energy. 2016. pp. 124–135. doi: 10.1016/j.apenergy.2016.06.061

[16] De Bok FAM, Van Leerdam RC, Lomans BP, Smidt H, Lens PNL, Janssen AJH, et al. Degradation of methanethiol by methylotrophic methanogenic archaea in a lab-scale upflow anaerobic sludge blanket reactor. Appl Environ Microbiol. 2006;72: 7540–7547. doi: 10.1128/AEM.01133-06 17012592 PMC1694231

[17] Dalby FR, Svane S, Sigurdarson JJ, Sørensen MK, Hansen MJ, Karring H, et al. Synergistic tannic acid-fluoride inhibition of ammonia emissions and simultaneous reduction of methane and odor emissions from livestock waste. Environ Sci Technol. 2020;Accepted. doi: 10.1021/acs.est.0c01231 32407626

[18] Dalby FR, Hansen MJ, Feilberg A, Kümmel S, Nikolausz M. Effect of tannic acid combined with fluoride and lignosulfonic acid on anaerobic digestion in the agricultural waste management chain. Bioresour Technol. 2020;307: 123171. doi: 10.1016/j.biortech.2020.123171 32203867

[19] Laukenmann S, Polag D, Heuwinkel H, Greule M, Gronauer A, Lelieveld J, et al. Identification of methanogenic pathways in anaerobic digesters using stable carbon isotopes. Eng Life Sci. 2010;10: 509–514. doi: 10.1002/elsc.201000074

[20] Mulat DG, Ward AJ, Adamsen APS, Voigt NV, Nielsen JL, Feilberg A. Quantifying contribution of synthrophic acetate oxidation to methane production in thermophilic anaerobic reactors by membrane inlet mass spectrometry. Environ Sci Technol. 2014;48: 2505–2511. doi: 10.1021/es403144e 24437339

[21] Dalby F, Fuchs A, Feilberg A. Methanogenic pathways and δ13C values from swine manure with a cavity ring-down spectrometer: Ammonia cross-interference and carbon isotope labeling. Rapid Commun Mass Spectrom. 2020;34: 1–13. doi: 10.1002/rcm.8628 31658498

[22] Ottosen LDM, Poulsen H V., Nielsen DA, Finster K, Nielsen LP, Revsbech NP. Observations on microbial activity in acidified pig slurry. Biosystems Engineering. 2009. pp. 291–297. doi: 10.1016/j.biosystemseng.2008.12.003

[23] Fuchs W, Wang X, Gabauer W, Ortner M, Li Z. Tackling ammonia inhibition for efficient biogas production from chicken manure: Status and technical trends in Europe and China. Renew Sustain Energy Rev. 2018;97: 186–199. doi: 10.1016/j.rser.2018.08.038

[24] Sun C, Cao W, Banks CJ, Heaven S, Liu R. Biogas production from undiluted chicken manure and maize silage: A study of ammonia inhibition in high solids anaerobic digestion. Bioresour Technol. 2016;218: 1215–1223. doi: 10.1016/j.biortech.2016.07.082 27474956

[25] Feilberg A, Liu D, Adamsen APS, Hansen MJ, Jonassen KEN. Odorant emissions from intensive pig production measured by online proton-transfer-reaction mass spectrometry. Environ Sci Technol. 2010;44: 5894–5900. doi: 10.1021/es100483s 20586445

[26] Cappellin L, Karl T, Probst M, Ismailova O, Winkler PM, Soukoulis C, et al. On Quantitative Determination of Volatile Organic Compound Concentrations Using Proton Transfer Reaction Time-of-Flight Mass Spectrometry. Environ Sci Technol. 2012;46: 2283–2290. doi: 10.1021/es203985t 22296026

[27] Ellis AM, Mayhew CA. Experimental: Components and Principles. Proton Transfer Reaction Mass Spectrometry Principles and Applications. John Wiley & Sons Ltd; 2013.

[28] Coplen TB, Brand WA, Gehre M, Gröning M, Meijer HAJ, Toman B, et al. New guidelines for δ13C measurements. Anal Chem. 2006;78: 2439–2441. doi: 10.1021/ac052027c 16579631

[29] Coplen TB. Guidelines and recommended terms for expression of stable-isotope-ratio and gas-ratio measurement results. Rapid Commun Mass Spectrom. 2011;25: 2538–2560. doi: 10.1002/rcm.5129 21910288

[30] Conrad R. Quantification of methanogenic pathways using stable carbon isotopic signatures: A review and a proposal. Org Geochem. 2005;36: 739–752. doi: 10.1016/j.orggeochem.2004.09.006

[31] Conrad R, Noll M, Claus P, Klose M, Bastos WR, Enrich-Prast A. Stable carbon isotope discrimination and microbiology of methane formation in tropical anoxic lake sediments. Biogeosciences. 2011;8: 795–814. doi: 10.5194/bg-8-795-2011

[32] Geng L, Savarino J, Caillon N, Gautier E, Farquhar J, Dottin JW, et al. Intercomparison measurements of two 33S-enriched sulfur isotope standards. Journal of Analytical Atomic Spectrometry. 2019. pp. 1263–1271. doi: 10.1039/c8ja00451j

[33] Ding T. Determination of the absolute 32S/34S and 32S/33S ratios of IAEA-S-1, IAEA-S-2 and IAEA-S-3 reference materials and V-CDT sulfur isotope standard. Chinese Sci Bull. 1998;43: 33.

[34] Mulat DG, Feilberg A. GC/MS method for determining carbon isotope enrichment and concentration of underivatized short-chain fatty acids by direct aqueous solution injection of biogas digester samples. Talanta. 2015;143: 56–63. doi: 10.1016/j.talanta.2015.04.065 26078128

[35] Koch FC, McMeekin TL. A new direct nesslerization micro-kjeldahl method and a modification of the nessler-folin reagent for ammonia. J Am Chem Soc. 1924;46: 2066–2069. doi: 10.1021/ja01674a013

[36] APHA/AWWA/WEF. Standard Methods for the Examination of Water and Wastewater. Stand Methods. 2012. ISBN 9780875532356

[37] van Dorst J, Bissett A, Palmer AS, Brown M, Snape I, Stark JS, et al. Community fingerprinting in a sequencing world. FEMS Microbiol Ecol. 2014;89: 316–330. doi: 10.1111/1574-6941.12308 24580036

[38] Vrieze J De, Ijaz UZ, Saunders AM, Theuerl S. Terminal restriction fragment length polymorphism is an “old school” reliable technique for swift microbial community screening in anaerobic digestion. 2018; 1–12. doi: 10.1038/s41598-018-34921-7 30429514 PMC6235954

[39] Wörner S, Pester M. The active sulfate-reducing microbial community in littoral sediment of oligotrophic lake constance. Front Microbiol. 2019;10. doi: 10.3389/fmicb.2019.00247 30814991 PMC6381063

[40] Karnachuk O V., Rusanov II, Panova IA, Grigoriev MA, Zyusman VS, Latygolets EA, et al. Microbial sulfate reduction by Desulfovibrio is an important source of hydrogen sulfide from a large swine finishing facility. Sci Rep. 2021;11: 1–11. doi: 10.1038/s41598-020-79139-8 34021225 PMC8140134

[41] Dar SA, Yao L, Van Dongen U, Kuenen JG, Muyzer G. Analysis of diversity and activity of sulfate-reducing bacterial communities in sulfidogenic bioreactors using 16S rRNA and dsrB genes as molecular markers. Appl Environ Microbiol. 2007;73: 594–604. doi: 10.1128/AEM.01875-06 17098925 PMC1796976

[42] Steinberg LM, Regan JM. Phylogenetic Comparison of the Methanogenic Communities from an Acidic, Oligotrophic Fen and an Anaerobic Digester Treating Municipal Wastewater Sludge. 2008;74: 6663–6671. doi: 10.1128/AEM.00553-08 18776026 PMC2576706

[43] Bühligen F, Lucas R, Nikolausz M, Kleinsteuber S. Anaerobe A T-RFLP database for the rapid pro fi ling of methanogenic communities in anaerobic digesters. Anaerobe. 2016;39: 114–116. doi: 10.1016/j.anaerobe.2016.03.013 27046270

[44] Klindworth A, Pruesse E, Schweer T, Peplies J, Quast C, Horn M, et al. Evaluation of general 16S ribosomal RNA gene PCR primers for classical and next-generation sequencing-based diversity studies. Nucleic Acids Res. 2013;41: 1–11. doi: 10.1093/nar/gks1039 22933715 PMC3592464

[45] Bolyen E, Dillon M, Bokulich N, Abnet C, Al-Ghalith G, Alexander H, et al. QIIME 2: Reproducible, interactive, scalable, and extensible microbiome data science. PeerJ Prepr. 2018. doi: 10.7717/peerj-cs.147 31341288 PMC7015180

[46] Callahan BJ, McMurdie PJ, Rosen MJ, Han AW, Johnson AJA, Holmes SP. DADA2: High-resolution sample inference from Illumina amplicon data. Nat Methods. 2016;13: 581–583. doi: 10.1038/nmeth.3869 27214047 PMC4927377

[47] McIlroy SJ, Saunders AM, Albertsen M, Nierychlo M, McIlroy B, Hansen AA, et al. MiDAS: The field guide to the microbes of activated sludge. Database. 2015;2015: 1–8. doi: 10.1093/database/bav062 26120139 PMC4483311

[48] R Core Team. A language and environment for statistical computing. R; 2017. Available: https://www.r-project.org/

[49] Eriksen J, Andersen AJ, Poulsen H V., Adamsen APS, Petersen SO. Sulfur turnover and emissions during storage of cattle slurry: Effects of acidification and sulfur addition. J Environ Qual. 2012;41: 1633. doi: 10.2134/jeq2012.0012 23099955

[50] Hansen MJ, Jonassen KEN, Løkke MM, Adamsen APS, Feilberg A. Multivariate prediction of odor from pig production based on in-situ measurement of odorants. Atmos Environ. 2016;135: 50–58. doi: 10.1016/J.ATMOSENV.2016.03.060

[51] Muyzer G, Stams AJM. The ecology and biotechnology of sulphate-reducing bacteria. Nat Rev Microbiol. 2008;6: 441–454. doi: 10.1038/nrmicro1892 18461075

[52] Khan MA, Patel PG, Ganesh AG, Rais N, Faheem SM, Khan ST. Assessing Methanogenic Archaeal Community in Full Scale Anaerobic Sludge Digester Systems in Dubai, United Arab Emirates. Open Microbiol J. 2018;12: 123–134. doi: 10.2174/1874285801812010123 29785219 PMC5960743

[53] Shin J, Cho SK, Lee J, Hwang K, Chung JW, Jang HN, et al. Performance and microbial community dynamics in anaerobic digestion of waste activated sludge: Impact of immigration. Energies. 2019;12: 1–15. doi: 10.3390/en12030573

[54] Petersen SO, Andersen AJ, Eriksen J. Effects of cattle slurry acidification on ammonia and methane evolution during storage. J Environ Qual. 2012;41: 88. doi: 10.2134/jeq2011.0184 22218177

[55] Eriksen J, Adamsen APS, Nørgaard J V., Poulsen HD, Jensen BB, Petersen SO. Emissions of sulfur-containing odorants, ammonia, and methane from pig slurry: effects of dietary methionine and benzoic acid. J Environ Qual. 2010;39: 1097. doi: 10.2134/jeq2009.0400 20400605

[56] Pinhal S, Ropers D, Geiselmann J, Jong H de. Acetate metabolism and the inhibition of bacterial growth by acetate. J Bacteriol. 2019;201: 1–19. doi: 10.1128/JB.00147-19 30988035 PMC6560135

[57] McCartney DM, Oleszkiewicz JA. Competition between methanogens and sulfate reducers: effect of COD:sulfate ratio and acclimation. Water Environ Res. 1993;65: 655–664. doi: 10.2175/wer.65.5.8

[58] Isa Z, Grusenmeyer S, Verstraete W. Sulfate Reduction Relative to Methane Production in High-Rate Anaerobic Digestion: Technical Aspects. Appl Environ Microbiol. 1986;51: 572–579. Available: http://www.ncbi.nlm.nih.gov/pmc/articles/PMC238921/pdf/aem00138-0124.pdf doi: 10.1128/aem.51.3.572-579.1986 16347018 PMC238921

[59] Dar SA, Kleerebezem R, Stams AJM, Kuenen JG, Muyzer G. Competition and coexistence of sulfate-reducing bacteria, acetogens and methanogens in a lab-scale anaerobic bioreactor as affected by changing substrate to sulfate ratio. Appl Microbiol Biotechnol. 2008;78: 1045–1055. doi: 10.1007/s00253-008-1391-8 18305937 PMC2271084

[60] de Jesus EB, de Andrade Lima LRP, Bernardez LA, Almeida PF. Inhibition of Microbial Sulfate Reduction By Molybdate. Brazilian J Pet Gas. 2015;9: 95–106. doi: 10.5419/bjpg2015-0010

[61] Stoeva MK, Coates JD. Specific inhibitors of respiratory sulfate reduction: Towards a mechanistic understanding. Microbiol (United Kingdom). 2019;165: 254–269. doi: 10.1099/mic.0.000750 30556806

[62] Brandt KK, Patel BKC, Ingvorsenl K. Desulfocella halophila ge. nov., sp. nov., a halophilic, fatty-acid-oxidizing, sulfate-reducing bacterium isolated from sediments of the Great Salt Lake. 2015; 193–200. 10.1099/00207713-49-1-193 10028263

[63] Whitehead TR, Spence C, Cotta MA. Inhibition of hydrogen sulfide, methane, and total gas production and sulfate-reducing bacteria in in vitro swine manure by tannins, with focus on condensed quebracho tannins. Appl Microbiol Biotechnol. 2013;97: 8403–8409. doi: 10.1007/s00253-012-4562-6 23149758

[64] Han W, He P, Lin Y, Shao L, Lü F. A methanogenic consortium was active and exhibited long-term survival in an extremely acidified thermophilic bioreactor. Front Microbiol. 2019;10: 1–15. doi: 10.3389/fmicb.2019.00001 32038509 PMC6988822

