# Supplementary material for: Effects of combined tannic acid/fluoride on sulfur transformations and methanogenic pathways in swine manure
Source: PLoS One. 2021 Sep 23;16(9):e0257759. doi: 10.1371/journal.pone.0257759 (PMC8459979; doi:10.1371/journal.pone.0257759)
Supplement: S1 Appendix — Production of methane (a), emission of carbon dioxide (b), δ13CCH4 values (c), and δ13CCO2 values (d-e) from degassed cattle manure treated with tannic acid and fluoride (TA-NaF) or acidified with sulfuric acid to around pH 5.5. (DOCX) [file pone.0257759.s001.docx]

**Appendix S1. Methanogenesis pathways in cattle manure – experiment 5**

**Methods**

Degassed cattle manure was collected from an anaerobic digester at Aarhus University Biogas Facility (Foulum, Denmark). The cattle manure was stored in 10 L closed plastic containers at 23 ºC for two days and sieved through 1.2 mm diameter pores. Samples for volatile fatty acid analysis were collected and stored at -18 °C until analysis. The pH of the bulk manure was measured with a pH-meter (Portamess 911, Knick, Germany). Hundred grams of sieved cattle manure was added to each of 16 x 100 mL DURAN reactors under agitation (MR 3001 K, Heidolph, Germany). This ensured even particle distribution in the manure while pouring it into the reactors. Subsequently, the manure reactors (DURAN reactors with manure) were treated with tannic acid (Merck, CAS 1401-55-4) and sodium fluoride (Merck, CAS 7681-49-4) to final concentrations of 7.5 mM and 1 mM, respectively, or acidified to pH 5.5 with a 1M sulfuric acid stock solution (AnalaR NORMAPUR, product 20700.420, 95%). Sodium acetate (Merck, CAS 127-09-3, anhydrous > 99%) and sodium acetate-2-^13^C (Merck, CAS 13291-89-9, 99 atom % ^13^C) were added as substrates from 1.67 M aqueous stock solutions. The cattle manure reactors were incubated at 23 ºC in a headspace gas monitoring setup (Fig 1b), which measured methane and carbon dioxide for 20 days.

**Results**

The estimated δ^13^C_2-C-Ac_ value was 27690 ± 818 (‰) based on the amount of added ^13^C-acetate and the amount of acetic acid measured in the unlabeled inoculum. A considerable amount of acidified cattle manure was lost upon acidification due to foaming caused by carbon dioxide degassing. This has decreased the production rate of methane and carbon dioxide but was not considered to affect the δ^13^C values. The δ^13^C_CH4_ and δ^13^C_CO2_ signatures of the untreated cattle manure at experiment start were -53.5 ± 0.6 ‰ and -13.1 ± 0.2 ‰, respectively. Fig S1 presents methane and carbon dioxide production and their respective δ^13^C values. Acidification treatment reduced methane production significantly by 61% (Fig S1a) and increased carbon dioxide emission by 234%. The effect on carbon dioxide emission is, however, also a function of a lower pH where more carbonate is dissociating into carbon dioxide. The pH of the acidified manures were 5.28, 5.60, and 2.76 just after acidification and this could explains the large uncertainty for the acidified manure bottles. The TA-NaF treatment also reduced methane production, but not with a statistically significant margin. Carbon dioxide emission was in general more than an order of magnitude higher than methane production for untreated and treated manures (Fig S1b). This suggests that carbon dioxide produced from fermentative pathways was not consumed by methanogens, which may not be adapted in relatively fresh manure [1]. Interestingly, TA-NaF treatment increased carbon dioxide emission significantly relative to the untreated controls, which may partly be explained by inhibition of hydrogenotrophic methanogenesis.


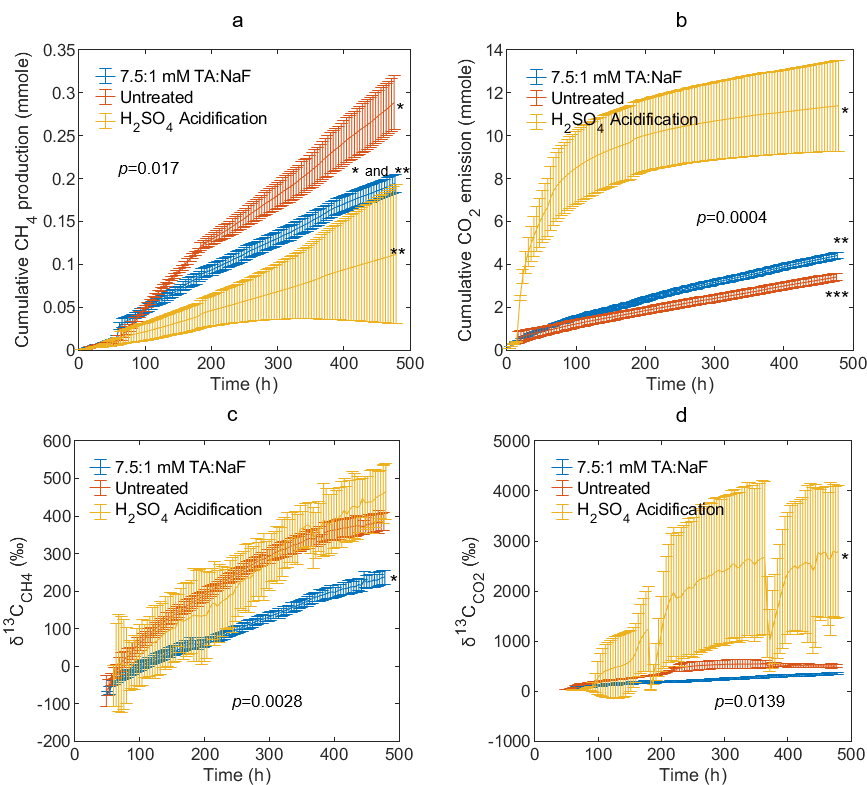


**Fig S1. Methanogenesis in cattle manure.** Production of methane (a), emission of carbon dioxide (b), δ^13^C_CH4_ values (c), and δ^13^C_CO2_ values (d-e) from degassed cattle manure treated with tannic acid and fluoride (TA-NaF) or acidified with sulfuric acid to around pH 5.5

When acetate is ^13^C-methyl labeled, acetoclastic methanogenesis activity results in ^13^C incorporation into methane, yielding relatively large δ^13^C_CH4_ values compared to δ^13^C_CO2_ than synthrophic acetate oxidation coupled to hydrogenotrophic methanogenesis (SAO-HM) activity would. The model predicted that close to 100% of the acetate derived methane came from SAO-HM derived methane for both untreated and TA-NaF treated cattle manure.

In Fig S1c and Fig S1d, the δ^13^C_CH4_ and δ^13^C_CO2_ values of TA-NaF treated manure followed a similar but delayed development compared to the untreated manure. In untreated manure, the δ^13^C_CO2_ values reached a plateau before the δ^13^C_CH4_ values. A similar tendency was observed in another labeling study [2], where the difference was attributed to quicker degassing of carbon dioxide relatively to methane, and hence an earlier δ^13^C_CO2_ peak. However, diffusion of methane is quicker than carbon dioxide due to its lower mass, and we believe another plausible explanation could be a continuous change in the relative activity between acetoclastic and hydrogenotrophic methanogens, which is related to different adaptation rates.

The δ^13^C_CO2_ values from acidified manure were extremely high (Fig S1e) and with large uncertainties. If assuming that only methanogenesis processes convert acetate, these values were not consistent with the theoretical maximum δ^13^C_CO2_ values considering the simultaneous low δ^13^C_CH4_ values. Sulfuric acid was used as acidification agent and consequently, sulfate concentrations were extremely high at 179.4 ± 11.6 mM and 176.4 ± 10.5 mM at day 2 and 21, respectively. Our data therefore strongly suggest that acetate was primarily consumed through sulfate reduction when the manure was acidified, as otherwise the δ^13^C_CO2_ values would be lower compared to δ^13^C_CH4_. Hence, it was difficult to conclusively determine whether acidification inhibited acetoclastic methanogenesis or SAO-HM.

1. Sommer SG, Clough TJ, Balaine N, Hafner SD, Cameron KC. Transformation of organic matter and the emissions of methane and ammonia during storage of liquid manure as affected by acidification. J Environ Qual. 2017;46: 514–521. doi:10.2134/jeq2016.10.0409

2. Laukenmann S, Polag D, Heuwinkel H, Greule M, Gronauer A, Lelieveld J, et al. Identification of methanogenic pathways in anaerobic digesters using stable carbon isotopes. Eng Life Sci. 2010;10: 509–514. doi:10.1002/elsc.201000074
