# Supplementary material for: Effects of combined tannic acid/fluoride on sulfur transformations and methanogenic pathways in swine manure
Source: PLoS One. 2021 Sep 23;16(9):e0257759. doi: 10.1371/journal.pone.0257759 (PMC8459979; doi:10.1371/journal.pone.0257759)
Supplement: S2 Appendix — Production of methane (a), emission carbon dioxide (b) from acidified and TA-NaF treated wastewater sludge. δ13CCH4 values (c) and δ13CCH4 values (d). (DOCX) [file pone.0257759.s002.docx]

### **Appendix S2. Methanogenesis pathways in wastewater sludge – experiment 6**

**Method**

### Wastewater sludge was collected from a biogas reactor at Egå Wastewater Treatment Plant, Denmark. The inoculum was filtered through a 0.8 mm mesh and diluted 1:1 (v:v) with demineralized water and then stored at 23 ºC for 4 months before experiment start. Samples for volatile fatty acid analysis and pH was measured as described in section 2.2.1. Approximately 50 g of wastewater sludge was added to 8 x 100 mL DURAN reactors under agitation (MR 3001 K, Heidolph, Germany). The wastewater sludge reactors were then treated with tannic acid with sodium fluoride to final concentrations of 5 mM and 1 mM, respectively, or acidified to pH 5.5 with a 0.5 M hydrochloric acid stock solution (Merck, CAS 1.00317, fuming 37%). Sodium acetate and sodium acetate-2-13C were added as described in section 2.2.1 and the reactors were incubated in the headspace gas monitoring setup at 23 ºC for 15 days (Fig 1b in main paper).

**Results**

In contrast to the swine and cattle manure, wastewater treatment plant sludge is often dominated by acetoclastic methanogens [1,2]. Consequently, wastewater sludge was incubated in a similar experimental setup as cattle manure to elucidate the influence of TA-NaF and acidification treatment on an inoculum expected to be dominated by acetoclastic methanogenesis. To prevent sulfate reduction from consuming the ^13^C-acetate, sulfuric acid was replaced with hydrochloric acid as acidification agent. The δ^13^C_2-C-Ac_ value in the wastewater sludge reactors were 5152 ± 355 ‰ as shown in Table 1 in the main paper. For the unlabeled reactors, the δ^13^C_CH4_ started at -35 ‰ and decreased to -68 ‰ by experiment end, whereas the δ^13^C_CO2_ was around -10 ‰ and decreased quickly to -22 ‰, where it remained until experiment end.

Fig S2a shows very little methane production with 5:1 mM TA-NaF treatment and large methane production from acidified wastewater sludge reactors when compared to the untreated control reactors at the end of the experiment. Each treatment was only done in duplicates, hence statistical significant difference in methane production was only seen between TA-NaF and HCl treatments. Carbon dioxide emission was less affected by treatments (Fig S2b). Acidified wastewater sludge labeled with ^13^C-acetate produced very high δ^13^C_CH4_ values, peaking at around 4000 ‰ and then slowly declining. Untreated wastewater sludge peaked at around 2400 ‰, whereas TA-NaF treatment produced methane with δ^13^C_CH4_ values between 0 and 200 ‰ (Fig S2c). The δ^13^C_CO2_ values (Fig S2d) were surprisingly small for all wastewater sludge reactors (between 0 and 200 ‰), which prove that the wastewater sludge was dominated by acetoclastic methanogenesis. When δ^13^C_CH4_ peaked, the δ^13^C_CO2_ values relative to δ^13^C_CH4_ values indicated that particularly acetoclastic methanogenesis was inhibited by TA-NaF. Acidification had the opposite effect and inhibited hydrogenotrophic methanogens. In Fig S2c there was a steady decline in the δ^13^C_CH4_ values after peak maximum, which suggest an increasing activity of hydrogenotrophic methanogens, but it could also be a consequence of ^13^C-labeled acetate depletion in the wastewater sludge. However, in the unlabeled control reactors there was a gradual decrease in the δ^13^C_CH4_ signatures, which is indicative of increased hydrogenotrophic methanogenesis [3].


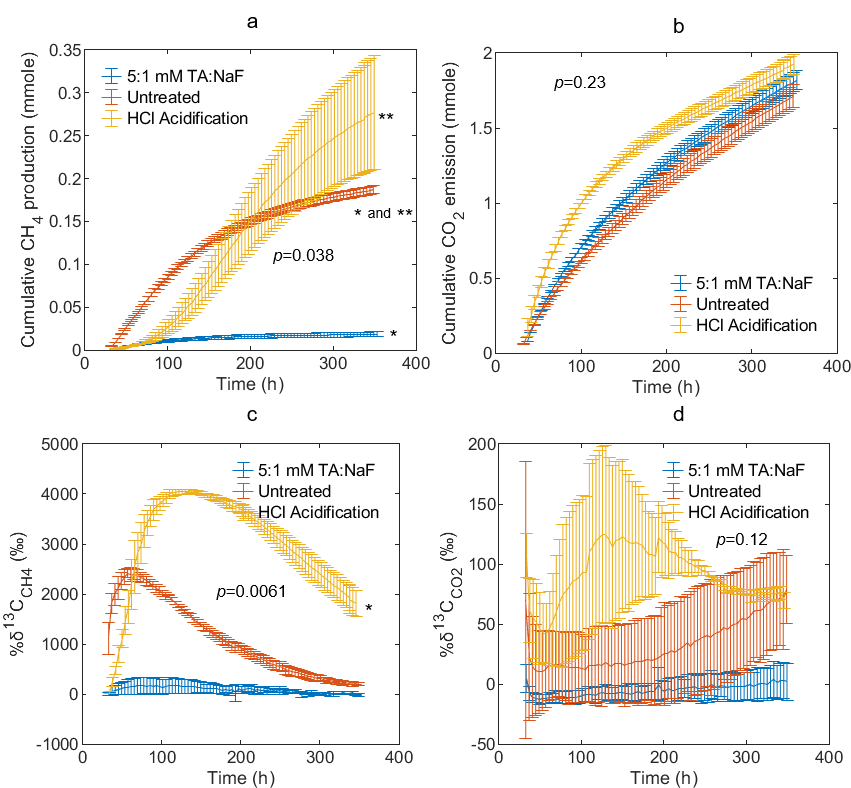


**Fig S2. Methanogenesis in wastewater sludge.** Production of methane (a) and carbon dioxide (b) from acidified and TA-NaF treated wastewater sludge. δ^13^C_CH4_ values (c) and δ^13^C_CH4_ values (d).

1. Khan MA, Patel PG, Ganesh AG, Rais N, Faheem SM, Khan ST. Assessing Methanogenic Archaeal Community in Full Scale Anaerobic Sludge Digester Systems in Dubai, United Arab Emirates. Open Microbiol J. 2018;12: 123–134. doi:10.2174/1874285801812010123

2. Shin J, Cho SK, Lee J, Hwang K, Chung JW, Jang HN, et al. Performance and microbial community dynamics in anaerobic digestion of waste activated sludge: Impact of immigration. Energies. 2019;12: 1–15. doi:10.3390/en12030573

3. Nikolausz M, Walter RFH, Sträuber H, Liebetrau J, Schmidt T, Kleinsteuber S, et al. Evaluation of stable isotope fingerprinting techniques for the assessment of the predominant methanogenic pathways in anaerobic digesters. Appl Microbiol Biotechnol. 2013;97: 2251–2262. doi:10.1007/s00253-012-4657-0
