# Supplementary material for: Effects of combined tannic acid/fluoride on sulfur transformations and methanogenic pathways in swine manure
Source: PLoS One. 2021 Sep 23;16(9):e0257759. doi: 10.1371/journal.pone.0257759 (PMC8459979; doi:10.1371/journal.pone.0257759)
Supplement: S3 Appendix — (a) NH4H2O+ cluster ions with and without a Nafion tube prior to the PTR-MS inlet from swine manure emitting ammonia at concentrations between 30 and 40 ppm (measured at a reduced electric field of 159 Townsend). In the upper right corner of (a) spectral overlap between NH4H2O+ and H233SH+ ions is observed at 5 ppm ammonia and 4–5 ppm hydrogen sulfide (measured at a reduced electric field of 152 Townsend). “Nafion” and “Background” in (a) indicate at what time a Nafion tube and a charcoal filter (reflecting background signal) were applied in the measurement setup, respectively. (b) Measured ion ratios corresponding to the m/z ratios were hydrogen sulfide and methanthiol isotopologues are measured (Nafion tube applied). Full black lines in (b) indicate theoretical m/z ratios of the [H3S]+ and [CH4SH]+ ions (measured at a reduced electric field of 152 Townsend). (DOCX) [file pone.0257759.s003.docx]

**Appendix S3. PTR-MS interference**

Initial test revealed that the high concentrations of ammonia emitted from the fresh swine manure and the high humidity at the PTR-MS inlet led to significant amounts of the NH_4_H_2_O^+^ cluster ion detected at *m/z* 36.045. At moderate to low hydrogen sulfide levels it was therefore crucial to eliminate this ion from forming to properly quantify the H_2_^33^SH^+^ ion signal at *m/z* 35.995. In Fig S3a, we measured the NH_4_H_2_O^+^ cluster ion with a Nafion tube prior to the PTR-MS inlet. The Nafion tube reduced NH_4_H_2_O^+^ ions by more than 99%, which was an indirect consequence of ammonia and water vapor removal. In the upper right corner of Fig S3a, raw measurements of gas from unlabeled swine manure in the *m/z* 36 region shows clear interference between the two ions, underlining the necessity of applying the Nafion tube in our experiments. In Fig S3b, hydrogen sulfide and methanethiol ratios were measured from unlabeled swine manure while applying the Nafion membrane tube prior to the PTR-MS inlet. The measured ion ratios were 0.88 ± 0.05% for *m/z* 36: *m/z* 35 (where hydrogen sulfide isotopologues were detected) and 2.13 ± 0.23% *m/z* 50: *m/z* 49 (where methanethiol isotopologues were detected). The *m/z* 50: *m/z* 49 ratio is approximately 1.1% higher due to contribution from ^13^C. The corresponding theoretical ion ratios are 0.826 ± 0.02% and 1.939 ± 0.153%, respectively, using error propagated standard deviations. The higher variation associated with the ratio between *m/z* 50 and *m/z* 49 was attributed to low signal strength at *m/z* 50, as a consequence of very limited methanethiol emissions.


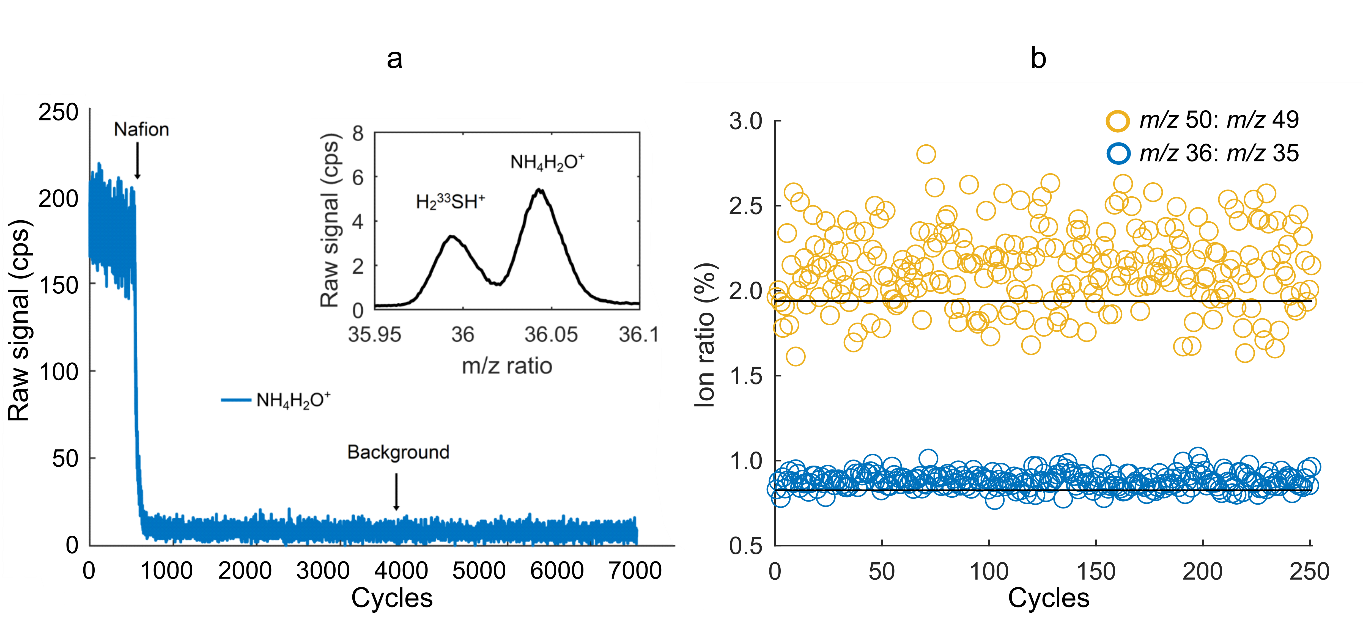


**Fig S3. PTR-MS interference.** (a) NH_4_H_2_O^+^ cluster ions with and without a Nafion tube prior to the PTR-MS inlet from swine manure emitting ammonia at concentrations between 30 and 40 ppm (measured at a reduced electric field of 159 Townsend). In the upper right corner of (a) significant spectral overlap between NH_4_H_2_O^+^ and H_2_^33^SH^+^ ions is observed at 5 ppm ammonia and 4-5 ppm hydrogen sulfide (measured at a reduced electric field of 152 Townsend). “Nafion” and “Background” in (a) indicate at what time a Nafion tube and a charcoal filter (reflecting background signal) were applied in the measurement setup, respectively. (b) Measured ion ratios corresponding to the m/z ratios were hydrogen sulfide and methanthiol isotopologues are measured (Nafion tube applied). Full black lines in (b) indicate theoretical m/z ratios of the [H3S]^+^ and [CH4SH]^+^ ions using a reference R(^33/32^S) equal to 0.00788 (measured at a reduced electric field of 152 Townsend).
