# Supplementary material for: Effects of combined tannic acid/fluoride on sulfur transformations and methanogenic pathways in swine manure
Source: PLoS One. 2021 Sep 23;16(9):e0257759. doi: 10.1371/journal.pone.0257759 (PMC8459979; doi:10.1371/journal.pone.0257759)
Supplement: S4 Appendix — Chemical characterization of the inocula used in this study. Uncertainty is presented as the standard deviation of triplicate samples. N/A denotes that the quantity was not measured. TS = Total solids, VS = Volatile solids, TAN = Total ammonia nitrogen, TN = Total nitrogen, and VFA = Volatile fatty acids. (DOCX) [file pone.0257759.s004.docx]

**Appendix S4.**

**Table S1. Manure characterization** Chemical characterization of the inocula used in this study. Uncertainty is presented as the standard deviation of triplicate samples. N/A denotes that the quantity was not measured. TS = Total solids, VS = Volatile solids, TAN = Total ammonia nitrogen, TN = Total nitrogen, and VFA = Volatile fatty acids.

|  | TS (%) | VS (%) | TAN (g/L) | TN (g/L) | VFA (g/L) | Sulfate (mM) | pH | Inoculum used (g) |
| --- | --- | --- | --- | --- | --- | --- | --- | --- |
| Swine manure for experiment 1  (methanogenesis pathways) | N/A | N/A | N/A | N/A | 2.7 ± 0.1 | 5.9 ± 0.2 | 7.89 | 100 |
| Swine manure for experiment 2 (reduced sulfur compound emissions) | 10.3 ± 1.1 | 8.8 ± 1.3 | <0.5 | 7.8 ± 2.4 | 2.2 ± 0.3 | 7.8 ± 0.2 | 6.55 | 40 |
| Swine manure for experiment 3 (sulfur transformation pathways) | 4.6 ± 0.1 | 3.5 ± 0.1 | 3.4 ± 0.1 | N/A | 10.8 ± 0.34 | 2.9 ± 0.1 | 7.38 | 40 |
| Swine manure for experiment 4 (microbial community structure) | 3.1 ± 0.1 | 2.0 ± 0.1 | 5.2 ± 0.9 | N/A | 1.8 ± 0.04 | N/A | 7.68 | 120 |
| Cattle manure for experiment 4 (microbial community structure) | 2.6 ± 0.2 | 2.0 ± 0.1 | 1.2 ± 0.2 | N/A | 2.2 ± 0.5 | N/A | 7.03 | 120 |
| Cattle manure for experiment 5 (methanogenesis pathways) | 4.3 | N/A | 0.8 ± 0.03 | 3.2 ± 0.2 | 0.67 ± 0.01 | 10.1 ± 0.9 | 8.11 | 100 |
| Wastewater sludge for experiment 6 (methanogenesis pathways) | 0.5 | N/A | 0.6 ± 0.01 | N/A | 1.4 ± 0.1 | <0.5 | 7.36 | 50 |
